# Chromosome-level genome assembly of bunching onion illuminates genome evolution and flavor formation in *Allium* crops

**DOI:** 10.1038/s41467-022-34491-3

**Published:** 2022-11-05

**Authors:** Nanqiao Liao, Zhongyuan Hu, Jinshan Miao, Xiaodi Hu, Xiaolong Lyu, Haitian Fang, Yi-Mei Zhou, Ahmed Mahmoud, Guancong Deng, Yi-Qing Meng, Kejia Zhang, Yu-Yuan Ma, Yuelin Xia, Meng Zhao, Haiyang Yang, Yong Zhao, Ling Kang, Yiming Wang, Jing-Hua Yang, Yan-Hong Zhou, Ming-Fang Zhang, Jing-Quan Yu

**Affiliations:** 1 Institute of Vegetable Science, Zhejiang University, 310058 Hangzhou, Zhejiang P. R. China; 2 Horticultural Institute of Science and Technology, Weifang University of Science and Technology, 262700 Weifang, Shandong P. R. China; 3 Novogene Bioinformatics Institute, 100083 Beijing, P. R. China; 4 Key Laboratory of Horticultural Plant Growth and Development, Ministry of Agriculture and Rural Affairs, 310058 Hangzhou, Zhejiang P. R. China; 5 Hainan Institute of Zhejiang University, Yazhou Bay Science and Technology City, 572025 Sanya, Hainan P. R. China; 6 Ningxia Key Laboratory for Food Microbial-applications Technology and Safety Control, School of Food & Wine, Ningxia University, 750021 Yinchuan, Ningxia P. R. China

**Keywords:** Plant genetics, Genomics, Agricultural genetics, Evolutionary genetics

## Abstract

The *Allium* genus is cultivated globally as vegetables, condiments, or medicinal plants and is characterized by large genomes and strong pungency. However, the genome evolution and genomic basis underlying their unique flavor formation remain poorly understood. Herein, we report an 11.27-Gb chromosome-scale genome assembly for bunching onion (*A. fistulosum*). The uneven bursts of long-terminal repeats contribute to diversity in genome constituents, and dispersed duplication events largely account for gene expansion in *Allium* genomes. The extensive duplication and differentiation of alliinase and lachrymatory factor synthase manifest as important evolutionary events during flavor formation in *Allium* crops. Furthermore, differential selective preference for flavor-related genes likely lead to the variations in isoalliin content in bunching onions. Moreover, we reveal that China is the origin and domestication center for bunching onions. Our findings provide insights into *Allium* genome evolution, flavor formation and domestication history and enable future genome-assisted breeding of important traits in these crops.

## Abstract (web summary)

Genome evolution of *Allium* genus and genomic basis underlying the pungency flavor formation remain poorly understood. Here, the authors assemble the genome of bunching onion and conduct population genetics analyses to reveal *Allium* genome evolution, flavor formation and domestication history.

## Introduction

The *Allium* genus is naturally distributed geologically in the northern hemisphere with a major center of diversity ranging from the Mediterranean Basin to Central Asia^1, 2^. It possesses strong adaptability to seasonal drought and harsh conditions. Awareness of the potential of *Allium* vegetables to enhance human health is driving the increasing demand for these functional crops. In addition, the *Allium* genus is notable for a unique set of sulfoxides derived from cysteine^3–5^, which contribute to their characteristic flavor, medicinal properties, disease resistance, and pest repellency^5–7^. Compared to quality-related components such as sugar, organic-acid, alkaloid-, and terpene-derived metabolites, less is known about the biosynthesis and regulation of *Allium*-specific alk(en)yl cysteine sulfoxides (ACSOs)^8–10^, such as alliin, isoalliin, and methiin. Among the major ACSOs in the *Allium* genus, isoalliin can be hydrolyzed to propanthial S-oxide (commonly known as lachrymatory factor) with the aid of alliinase (ALL) and lachrymatory factor synthase (LFS)^3^, whereas alliin can be spontaneously catalyzed into allicin upon tissue damage^3, 4, 11^. However, the genomic basis of this unique flavor formation in *Allium* crops remains unclear.

The *Allium* genus possesses some major cash crops including bunching onions, Welsh onions or storey onions (*A. fistulosum*), bulb onions (*A. cepa*), garlic (*A. sativum*), and shallots (*A. ascalonicum*). Owing to their morphological resemblance, weak reproductive isolation, and similar chromosome karyotypes, the genetic relationships among *Allium* species are not well defined^2, 12^. Traditional *Allium* taxonomy is mostly based on plant morphology^9, 13^. For instance, *A. fistulosum* is generally classified based on its tillering characteristics and pseudo-stem morphology. Shallots (*A. ascalonicum*) are often mistaken for bunching onions because of their similar tillering characteristics. Chinese red onions (*A. cepa* var. *proliferum*) are often confused with storey onions (*A. fistulosum* L. var*. viviparum*), because of their similar aerial bulbs. These ambiguities in *Allium* taxonomy often leads to misunderstanding of the evolution and domestication in this genus, which needs to be clarified at the genomic level.

Natural or artificial interspecific hybridizations are ubiquitous among the *Allium* genus, which have been empirically and randomly utilized to introduce disease resistance and quality traits^14–17^. Genome-informed interspecific hybridization could be an effective strategy to expand *Allium* genetic diversity for desirable agronomic traits. However, the *Allium* genera are notorious for their large genomes with a high proportion of repetitive sequences, which consequently lead to lack of high-quality genome assemblies in this genus^18, 19^. As a result, genome-assisted breeding of these important crops lags that of crops with relatively small genomes. Therefore, genomic information is highly demanded and desirable for *Allium* research and breeding communities.

Here, we report a chromosome-scale reference genome assembly of *A. fistulosum* obtained by combining PacBio, Bionano, HiC, and Illumina sequencing technology toolkits. Comparative genomics and genome collinearity analyses have robustly illuminated the genome evolution and relatedness in *Allium* crops. In addition, we investigate the evolution of genes involved in the biosynthesis and hydrolysis of ACSOs to elucidate the genomic mechanisms underlying the special flavor formations in *Allium* crops. Furthermore, 135 *Allium* accessions are re-sequenced to clarify the phylogenomic evolution and migration routes of the *Allium* crops. Additionally, transcriptomic and metabolic analyses are performed to explore the formation of sulfur-containing flavors in *Allium* crops. The present study provides insights into genome evolution and expansion of the *Allium* species and will enable genomic-aided breeding.

## Results

### Chromosome-scale mega-genome assembly and gene annotations

**Fig. 1:**
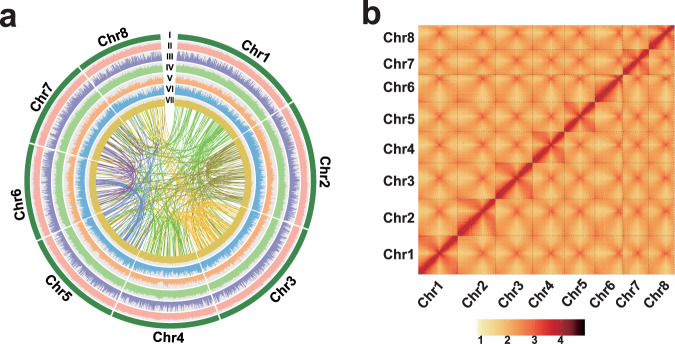
High-quality genome assembly of the *A. fistulosum*. **a** The rings indicate eight chromosomes (I), TE density (II), gene density (III), *Gypsy* retrotransposons density (IV), *Copia* retrotransposons density (V), Long interspersed nuclear elements density (VI), GC contents (VII), and inner lines indicate syntenic blocks. **b** Genome-wide chromatin interactions of eight chromosomes.

We assembled a chromosome-level genome of the bunching onion (*A. fistulosum*) using de novo genome sequencing of the bunching onion landrace. First, the *k-mer* analysis (*k* = 17) revealed an estimated genome size of 11.97 Giga base-pairs (Gb), a heterozygosity rate of 0.64%, and the repetitive sequences accounted for 89.89% of the whole genome (Supplementary Fig. 1 and Supplementary Table 2). PacBio sequences (1,649.82 Gb, 138.1 × genome coverage) were generated to assemble the genome into contigs, yielding a draft assembly of 27,972 contigs with a total length of 15.36 Gb and contig N50 of 4.72 mega base-pairs (Mb, Supplementary Table 1). After polishing and quality improvement, an improved assembly (scaffold N50 of 8.98 Mb) was obtained (Table 1 and Supplementary Table 3). Finally, Hi-C interaction datasets (1,136.4 Gb, 95.1 × genome coverage) were performed to construct the genome into super-scaffolds, and 98.71% of the assembled contigs were anchored into eight chromosomes (Fig. 1b and Supplementary Table 1, 3–5). The final genome assembly was 11.27 Gb with a contig N50 of 7.34 Mb and a super scaffold N50 of 1.34 Gb (Fig. 1, Table 1, and Supplementary Tables 3-4), which represents the best contiguity among the currently released *Allium* genome assemblies.

**Table 1:** The main genome assembly features of three major *Allium* crops

Assembly feature	*A. fistulosum*	*A. sativum*^11^	*A. cepa* ^a^ ^25^
Genome size	11,274 Mb	16,243 Mb	14,938 Mb
Super scaffold N50	1386 Mb	1691 Mb	—
Scaffold N50	8.98 Mb	725 kb	460 Kb
Contig N50	7.34 Mb	194 kb	48.29 Kb
Total length of transposable elements	7410 Mb	14,788 Mb	—
Gene number	62,259	57,561	86,073
Average gene length	5002.2 bp	5202.8 bp	—

^a^The information of *Allium cepa* genome was updated according to https://www.oniongenome.wur.nl/ (visiting date: 20220804).

Multiple genome assessments supported the high quality of *A. fistulosum* genome assembly. First, Benchmarking Universal Single-Copy Orthologue (BUSCO, 91.0% of the 1614 core eukaryotic genes) and Core Eukaryotic Gene Mapping Approach (CEGMA, 94.35% of conserved genes) analyses indicated the high completeness of the assembled genome (Supplementary Tables 6, 7). Meanwhile, the consensus quality value (QV) and the completeness of the *A. fistulosum* genome were 37.06% and 93.61%, respectively, indicating the high accuracy of our assembly^20^. By mapping short Illumina reads to the genome assembly, we obtained a mapping rate of 99.65%, a genome coverage of 99.34%, and an SNP rate of 0.0063%, demonstrating the high quality of the assembled genome (Supplementary Table 8). In addition, we observed good collinear relationships between the previous genetic map^21^ and the assembled chromosomes (Supplementary Fig. 2), which further supported our high-quality genome assembly. Regardless of the large genome size, the high long-terminal repeat retrotransposon assembly index (LAI, 24.81) suggested the gold continuity of the *A. fistulosum* genome assembly^22^.

A total of 7885 Mb (69.81% of the total genome) of repetitive sequences was annotated using RepeatMasker, TRF, and RepeatProteinMask (Supplementary Table 9). Most of the repetitive sequences were transposable elements (TEs), whereas long-terminal repeat (LTR) retrotransposons were the most abundant TEs in the *A. fistulosum* genome (62.18% of the total genome; Supplementary Fig. 3a, Supplementary Table 10). Complementary methods using de novo gene prediction, protein-based homology searches, and transcriptome-based predictions have been used to annotate protein coding sequences. In total, 62,255 genes were predicted, with an average gene length of 5000.13 base pairs (bp), an average gene coding sequence length of 820.11 bp, an average of 3.93 exons per gene, an average exon length of 208.47 bp, and a gene annotation rate of 98.30% (Supplementary Tables 11–13). Moreover, approximately a quarter (14,862 of 62,259) of the predicted genes were inserted by or overlapped with LTRs (Supplementary Fig. 3b). It has been reported that LTR in/around genes provide potential for their transduction, duplication and recombination, as well as alternative splicing and epigenetic control^23, 24^. Thus, we hypothesized that gene duplication or movement in *A. fistulosum* might occur along with the proliferation and transposition of LTRs. We also identified 1361 miRNA, 4365 tRNA, 2832 rRNA, and 3444 snRNAs in the *A. fistulosum* genome using the tRNAscan-SE pipeline (Supplementary Table 14).

### *Allium* genome evolution and chromosome homology

**Fig. 2:**
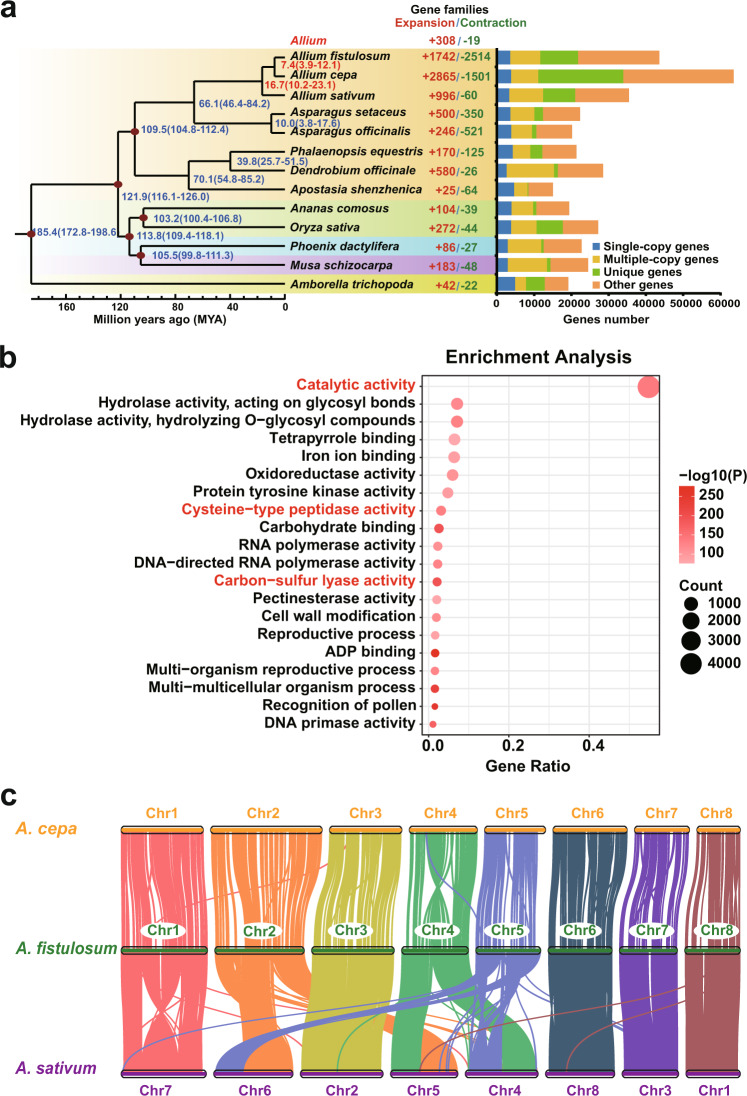
Genome evolution and relatedness of *Allium* crops. **a** The phylogenetic tree of 13 plant species and the evolution of the gene families. Numerical values besides each node show the estimated divergence time of each node. The red dots on the nods indicate the existence of fossil evidence that supported the estimated time. The numbers in the middle panel indicated the expanded and contracted gene families. The right panel displays the gene number of single-copy, multiple-copy, unique and other orthologues. **b** The top-20 enriched Gene Ontology terms of the shared expanded genes in three *Allium* crops. Chi-square test was used to calculate the *P*-values of the gene terms when all the expected frequency were higher than five, otherwise, we used Fisher’s exact test to calculate the *P*-values. **c** Genomic collinearity between three *Allium* crops. Source data are provided as a Source Data file.

To explore the genomic evolution of *Allium*, we selected 13 species from *Asparagale*, *Arecales*, *Poales*, *Amborellaceae*, and *Scitamineae* for further comparative genomic analysis. Among these plants, we discovered that the three *Allium* crops had the highest number of total and unique gene families (Fig. 2a). In total, 492 single-copy gene families were selected to reconstruct a maximum-likelihood (ML) tree, which illustrated that *A. fistulosum* and *A. cepa* diverged about 7.4 million years ago (MYA), and the common ancestor of *A. fistulosum* and *A. cepa* diverged from *A. sativum* about 16.7 MYA^11, 25^ (Fig. 2a). Among the selected genomes, the *Allium* crops exhibited numerous expanded and contracted genes, and 308 gene families were commonly expanded in all three *Allium* crops. Importantly, we found these gene families predominately enriched in gene ontology (GO) pathways like “Catalytic activity,” “Carbon-sulfur lyase activity,” and “Cysteine-type peptidase activity,” implying that the high sulfur-metabolism activities of *Allium* crops are responsible for the biosynthesis of sulfur compound related to flavor formation (Fig. 2a, b).

The distribution of *Ks* values of homologous pairs in the syntenic regions of *A. fistulosum* and *A. sativum* was similar to those of *A. sativum* and *Asparagus officinalis*^11^ (Supplementary Fig. 4), indicating that, similar to garlic, *A. fistulosum* also underwent three WGD events. In addition, most of the genes of the three major *Allium* crops corresponded to a single block in the collinearity analysis. Therefore, no unique WGD event occurred among these three genomes after divergence (Supplementary Fig. 5). Previously, a high level of macro-synteny between *A. fistulosum* and *A. cepa* has been reported using 103 anchor markers^21^. Here, we observed an almost one-to-one syntenic relationship at the chromosome level, except for Chr4, which underwent a clear inversion between the two *Allium* crops (Fig. 2c, Supplementary Fig. 6). In addition, four *A. fistulosum* chromosomes (Chr3, Chr6, Chr7, and Chr8) exhibited nearly one-to-one syntenic relationships with those of *A. sativum* (Chr2, Chr8, Chr3, and Chr1; Fig. 2c, Supplementary Fig. 6). The remaining four chromosomes exhibited several rearrangements. In details, a clear inversion was observed between Chr1 of *A. fistulosum* and Chr7 of *A. sativum*, Chr2 of *A. fistulosum* was partly syntenic to Chr5 and Chr6 of *A. sativum*, Chr4 of *A. fistulosum* showed partially syntenic relationships with Chr4 and Chr5 of *A. sativum*, and Chr5 of *A. fistulosum* was partially rearranged from Chr4 and Chr6 of *A. sativum* (Fig. 2c, Supplementary Fig. 6). Even though there were significant gaps among their genome sizes, high chromosome relatedness was observed among the three *Allium* genomes, which indicated that they were evolutionarily close to each other.

### Insights into genome expansions and gene duplications

**Fig. 3:**
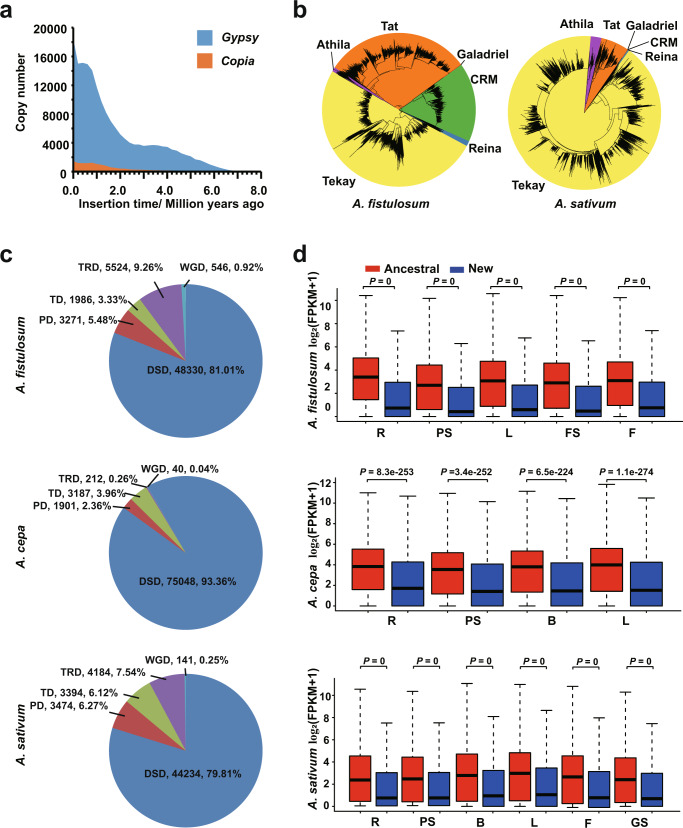
Extensive genome expansions and gene duplications in *Allium* crops. **a** Distribution of insertion times of *Gypsy* and *Copia* retrotransposons in *A. fistulosum*. **b** The phylogenetic relationships of *Gypsy* LTR-retrotransposons identified in *A. fistulosum* (left) and *A. sativum* (right) genomes. **c** The distribution of duplicated genes in three *Allium* genomes. DSD dispersed duplication, PD proximal duplication, TD tandem duplication, TRD transposed duplication, WGD whole-genome duplication. **d** The comparison of the expression levels of ancestral and new DSD genes in three *Allium* genomes (gene numbers: *n* = 13,676; *A. fistulosum*, *n* = 8424; *A. cepa*, and *n* = 17,817 *A. sativum*). In the box plots, the minima, maxima, center bounds of box showed 25%, 75% quartile values and the average values, while the whiskers showed maxima and minima values of expression levels. *P-*values were calculated using the two-tailed Wilcoxon test. R roots, PS pseudo-stems, B bulbs, L leaves, FS floral stalks, F flowers, GS garlic sprout. Source data are provided as a Source Data file.

*Allium* plants have long been known for their large genomes, which are among the largest in vegetable crops^26^. Therefore, we analyzed the key factors potentially associated with *Allium* genome expansion. We identified LTRs as the leading genomic constituents in the *A. fistulosum* (Supplementary Fig. 3a and Supplementary Table 10) and *A. sativum* genomes^10^. *Gypsy* was the predominant LTR and burst mostly within the last 2 million years (Fig. 3a and Supplementary Fig. 7). Within *Gypsy*, the Tekey and Tat clades were the most abundant LTRs in both *A. fistulosum* and *A. sativum* genomes. The CRM clade contributed significantly (10.38%) to the LTR components only in *A. fistulosum* (Fig. 3b). Although *Copia* LTRs comprised a small proportion of repeat sequence in both crops, their components significantly differed between the *A. fistulosum* and *A. sativum* genomes (Fig. 3a, Supplementary Figs. 7 and 8). Therefore, the accumulation of LTRs, especially the *Gypsy*-type, is responsible for genome enlargement in *Allium* crops, while uneven expansions of different LTR clades lead to diversity in *Allium* genome constituents.

The three *Allium* crops showed the highest number of total and unique gene families among the 13 selected genomes (Fig. 2a). To this end, we investigated the global landscape of gene duplications in 16 species with genome sizes ranging from 0.2 to 28 Gb. The large genomes had a relatively high frequency of dispersed duplication (DSD) events, demonstrating that DSD events play an important role in the gene expansion of large genomes (Fig. 3c, Supplementary Fig. 9). After classifying the duplicated paralogs into ancestral and new DSD genes based on synteny^27^, we identified 13,676, 17,817, and 8424 gene pairs (ancestral/new) in *A. fistulosum*, *A. sativum*, and *A. cepa*, respectively. The ancestral DSD genes exhibited significantly higher expression levels than the new genes (Fig. 3d). The expression bias between ancestral and new DSD genes might result from their different environmental responses or functional redundancies, which also implies the possible pseudogenization, sub-functionalization, or neo-functionalization after gene duplications. We propose that ubiquitous DSD events are responsible for gene expansion and divergence in the *Allium* genomes.

### Expansions and differentiations of flavor-related genes

**Fig. 4:**
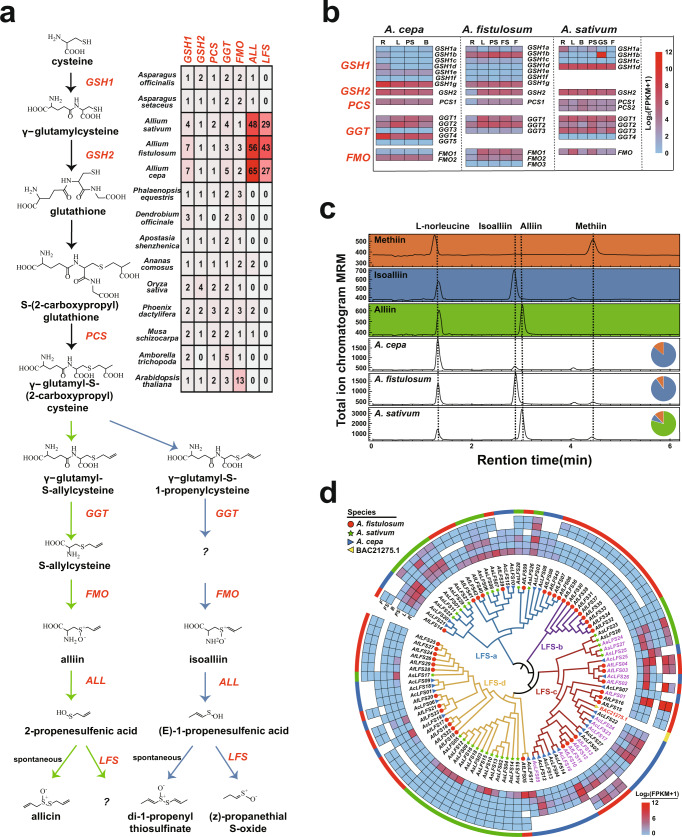
Massive duplication and differentiation of sulfur-related genes in *Allium*. **a** The biosynthesis and hydrolysis pathway of ACSOs and the number of each flavor- related gene in the 14 genomes. The pathway in blue relates to the biosynthesis of isoalliin that dominants in bulb onion and bunching onion. The pathway in green relates to the biosynthesis of alliin that dominants in garlic. **b** Expression patterns of ACSOs biosynthesis genes were investigated in three *Allium* crops. R roots, L leaves, PS pseudo-stems, B bulbs, FS floral stalks, F flowers, GS garlic sprout. The gene details are listed in Supplementary Data 1–3. **c** Quantification of ACSOs among three *Allium* crops by ultra-high-performance liquid chromatography. The pie chart exhibited the percentages of three major ACSOs (alliin, isoalliin, and methiin). **d** The phylogenetic tree and the expression pattern of LFS gene families from *A. fistulosum*, *A. cepa*, and *A. sativum* genomes. The outer rings highlight the genes from *A. fistulosum* (red color), *A. cepa* (blue color), and *A. sativum* (green color). The heatmap exhibited the gene expressions in different tissues: R roots, L leaves, PS pseudo-stems, B bulbs, FS floral stalks, F flowers. Source data are provided as a Source Data file.

To explore the evolutionary events of the putative genes involved in *Allium* flavor formation, we identified all ACSO-related genes in the 14 plant genomes (Fig. 4a). Seven γ-glutamylcysteine synthetase (*GSH1*) orthologs, one glutathione synthetase (*GSH2*) ortholog, one phytochelatin synthase (*PCS*) ortholog, three *γ-*glutamyl transpeptidase (*GGT*) orthologs, and three flavin-containing monooxygenase (*FMO*) orthologs were identified in the *A. fistulosum* genome (Fig. 4a). Among these genes, *AfGSH1b, AfGSH1g, AfGSH2, AfPSC1, AfGGT1, AfGGT2, AfFMO1*, and *AfFMO2* were constitutively expressed in the roots, pseudo-stems, leaves, floral stalks, and flowers, indicating that these genes might play roles in ACSOs biosynthesis in *Allium fistulosum* (Fig. 4b). The ALL gene encoding a key enzyme for ACSOs hydrolysis was found to be extensively expanded in bunching onion (56 *AfALL*s), bulb onion (65 *AcALL*s), and garlic (48 *AsALL*s) genomes^3^ compared with other selected genomes (Fig. 4a, Supplementary Fig. 10). Importantly, 43, 27, and 29 *LFS* orthologs were identified in the genomes of *A. fistulosum*, *A. cepa*, and *A. sativum*, respectively, whereas no *LFS* genes were found in species other than *Allium* among the selected genomes (Fig. 4a, d).

According to our ACSOs contents analysis and relevant report^4^, isoalliin was the major sulfur-containing bioactive substance in both *A. fistulosum* and *A. cepa*, whereas alliin was not detected in these two crops. However, alliin was the dominant sulfoxide in *A. sativum* and a small amount of isoalliin was detected (Fig. 4c). LFS is the key enzyme that catabolizes hydrolyzed isoalliin into the characteristic propanthial S-oxide (lachrymatory factor) in *Allium* crops. We classified the LFS gene family into four groups, LFS-a, LFS-b, LFS-c, and LFS-d, according to their phylogenetic structure annotated in three major *Allium* crops (Fig. 4d). In addition, *BAC21275.1* was verified as a functional LFS in bulb onions^28^ and was clustered into the LFS-c family (Fig. 4d). *AfLFS01*, the best hit with *BAC21275.1* in bunching onions, exhibited the highest transcriptional levels in most tissues, except roots, among all the *AfLFS* genes examined (Fig. 4d). Importantly, eight *AfLFS*, seven *AcLFS* and three *AsLFS* genes in this group presented similar expression patterns (Fig. 4d). Based on the close phylogenetic relationships with *BAC21275.1* and their similar expression patterns, we suppose that the LFS-c group might play synchronized roles in ACSOs hydrolysis in *Allium* crops. However, as the phylogenetically closest duplications, *AfLFS15* and *AfLFS16* showed different expression patterns against *AfLFS01* (Fig. 4d), suggesting the potential for functional differentiation of these homologous genes. We also observed different expression biases of the LFS groups in these crops. *A. fistulosum* and *A. cepa* preferred to extensively express LFS-c group members, whereas *A. sativum* predominately expressed LFS-a group members (Fig. 4d). Similarly, three proximally duplicated genes (*AfALL47*, *AfALL48*, and *AfALL49*) in Chr4 of bunching onion, four AcALL genes (*AcALL05*, *AcALL08*, *AcALL09*, and *AcALL47*) and four tandemly duplicated AsALL genes (*AsALL31*, *AsALL32*, *AsALL33*, and *AsALL34*) belong to the same cluster as known ALL genes (*AAD26853.1*, *AAA32639.1*, *AAB32477.1*, and *ACN78838.1*; Supplementary Fig. 10), all of which exhibited high expression levels in most tissues. These findings suggested that this gene cluster may be functional ALL genes in these three crops.

**Fig. 5:**
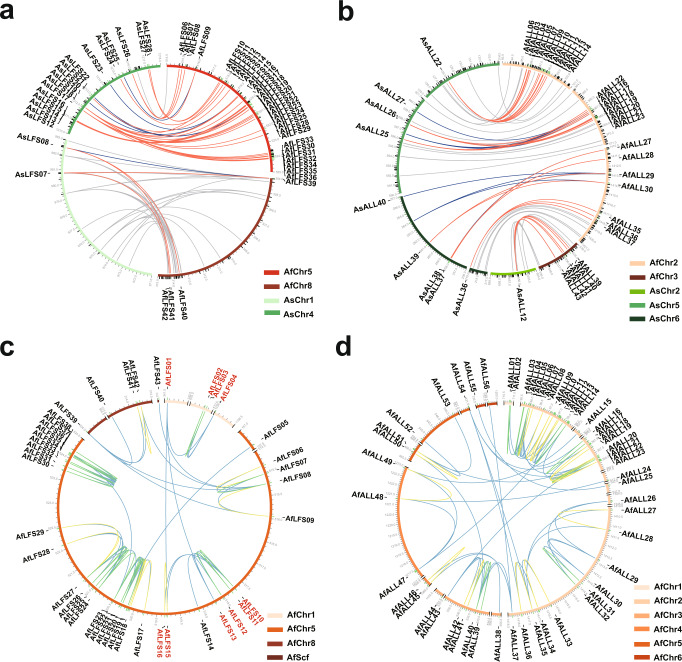
Independent expansions of *LFS* and *ALL* gene families between bunching onion and garlic. **a**, **b** The micro-collinearity relationship of *LFS* (**a**) and *ALL* (**b**) in *A. fistulosum* and *A. sativum*. The red lines indicate genes were expanded in *A. fistulosum*, the blue lines indicate genes were expanded in *A. sativum*, and the gray lines indicate other genes which show collinearity relationships. **c**, **d** The gene duplication relationships of *LFS* (**c**) and *ALL* (**d**) in *A. fistulosum*. The blue line indicates dispersed duplication relationship, the green line indicates tandem duplication relationship, and the yellow line indicates proximal duplication relationship. The gene details are listed in Supplementary Data 1–3.

The LFS-b group consisted of nine AfLFS genes that exhibited extremely low gene expression in all tissues (Fig. 4d). In contrast, 12 AsLFS genes clustered in an independent clade in the LFS-d group and were mainly expressed in the roots (Fig. 4d). Furthermore, the micro-collinearity analysis indicated that several *LFS* and *ALL* genes in bunching onion and garlic exhibited reciprocal one-to-many relationships (Fig. 5a, b). Thus, we inferred that the independent expansion and differentiation of *LFS* and *ALL* gene families after the divergence of bunching onion and garlic from their common ancestor might relate to their flavor diversity (Figs. 4d, 5a, b and Supplementary Fig. 10). Notably, the dispersed duplication, tandem duplication, and proximal duplication events of the *LFS* and *ALL* gene families were attributed to gene duplications (Fig. 5c, d, Supplementary Data 1). In particular, 33 *AfLFSs*, among 43 duplicates, were clustered in the form of tandem repeats within a 10 Mb region on Chr5 and the intertwined coexistence of LTRs and LFS genes in this region suggested that LTRs likely contribute to LFS gene family proliferation (Fig. 5c, Supplementary Fig. 11). We concluded that the independent expansion and differentiation of sulfur-metabolism-related genes prevail in *Allium* genomes, which might relate to the characteristic pungent flavor evolution in *Allium* crops.

### Population structure and migration routes of *A. fistulosum*

**Fig. 6:**
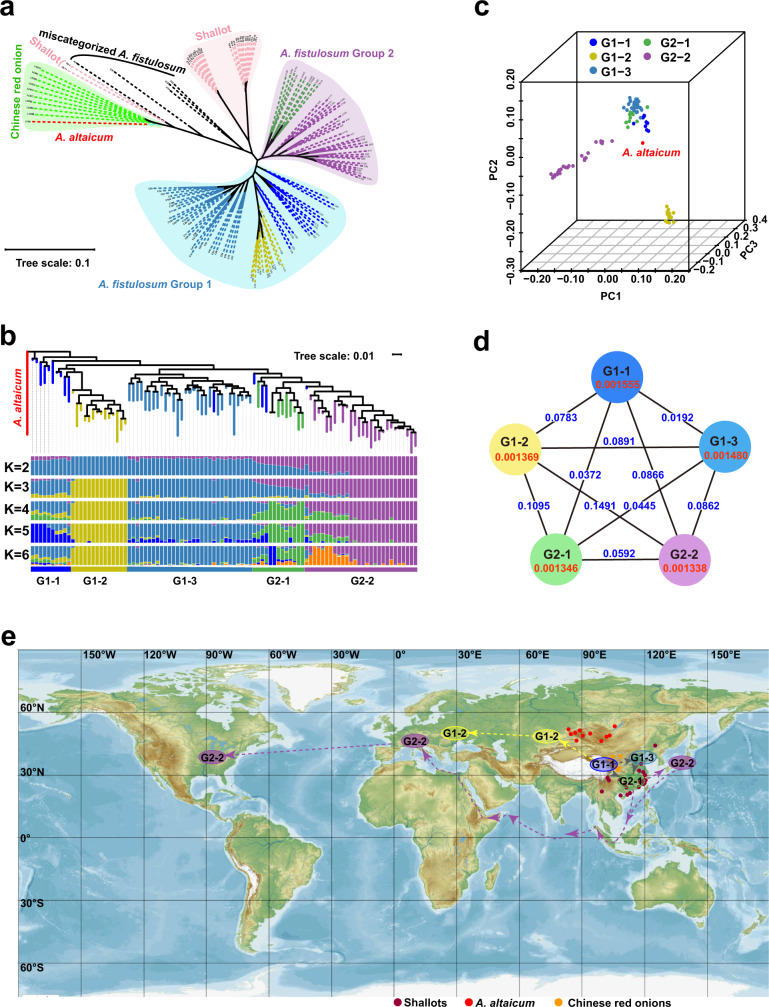
Population structure and the plausible migration routes of *A. fistulosum*. **a** The maximum-likelihood tree of 135 re-sequenced *Allium* accessions. **b** The maximum-likelihood tree and population structure of 96 re-sequenced *A. fistulosum* accessions **c**, The principal component analysis of 96 re-sequenced *A. fistulosums* accessions. **d** Nucleotide diversity (π) and population divergence (*F*_*ST*_) across the five subgroups. The numbers in red font were π values and the numbers in blue font were *Fst* values. **e** The possible migration routes for bunching onion (the map was downloaded from Mapswire, https://mapswire.com/world/physical-maps/). The dots indicated the habitat of the close relatives of *A. fistulosum*.

We re-sequenced 135 diverse *Allium* accessions to elucidate the phylogenetic relationships of *A. fistulosum* crops and their close relatives (Supplementary Figs. 12–13 and Supplementary Data 4). We generated 16,453.99 Gb Illumina short-reads with an average depth of 9.49 × genome coverage. In total, 48,218,339 SNPs and 18,182,723 insertions and deletions (InDels) with an average of 4.08 SNPs and 1.54 InDels per kb were identified. The maximum-likelihood tree of 135 *Allium* accessions revealed four main clusters centered on Chinese red onions (*A. cepa* var. *proliferum*), shallots (*A. ascalonicum*), *A. fistulosum* Group 1, and *A. fistulosum* Group 2 (Fig. 6a). All nine Chinese red onions and the *A. altaicum* accession clustered tightly into one group. Twenty-two shallots clustered together and displayed two distinct subgroups, whereas the remaining two shallots were close to the Chinese red onion group (Fig. 6a). Chinese red onions and shallots clustered apart from *A. fistulosum*, fitting well with their relatively low genome mapping rate (79–89%) to our reference genome. Therefore, we concluded that Chinese red onions and shallots are close relatives or sibling species of *A. fistulosum*. Ninety-six *A. fistulosum* accessions were clustered into two groups; the remaining five *A. fistulosum* accessions were clustered with shallots and Chinese red onions in the cladogram, which was indicative of ambiguity or mis-assignment in the *Allium* taxonomy (Fig. 6a and Supplementary Data 4).

To clarify the evolution and migration of *A. fistulosum*, we performed phylogenetic relationships and population structure analyses with 96 affirmed *A. fistulosum* accessions, using *A. altaicum* as an outgroup. The population structure of *A. fistulosum* was investigated and the cross validation (CV) error was minimized when *K* = 5 (Supplementary Fig. 14). The 96 *A. fistulosum* accessions could be categorized into five subgroups and fell into two groups (Fig. 6a, b). Group 1 (G1) could be divided into three subgroups with distinctive geographical distributions based on the population structure. G1-1 accessions were mainly distributed in western China, G1-2 accessions were distributed in Middle Asia and the Russian Federation, and G1-3 accessions were distributed in the northern and northeastern areas of China. Group 2 (G2) also displayed two differentiable geographic subgroups, with accessions from southeastern China clustered in subgroup G2-1. Accessions distributed in Japan, America, and Europe showed high genomic similarities and were clustered as G2-2 (Fig. 6b, e).

Furthermore, the principal components analysis (PCA) revealed that G1-2 and G2-2 clustered individually, whereas the three China-originated groups (G1-1, G1-3, and G2-1) showed a closer relationship (Fig. 6c). G1-3 possessed the lowest linkage disequilibrium (LD) decay, indicating that north and northeast China might be a diversity center of *A. fistulosum* (Supplementary Fig. 15). In contrast, G1-2 showed a unique structural pattern (when *K* = 3, 4, 5, and 6) with relatively low nucleotide diversity (π) and high LD decay (Fig. 6b, d and Supplementary Fig. 15). Furthermore, the fixation indexes (*F*_*ST*_) of G1-2/G1-1 (0.0783), G1-2/G1-3 (0.0891), G1-2/G2-1 (0.1095), and G1-2/G2-2 (0.1491) were higher than those of other subgroup pairs (Fig. 6d). The long leading branch of G1-2 (Fig. 6b) suggested that there might be an early divergence and independent domestication of G1-2 group, which also partly explains the relatively independent PCA clustering of G1-2 (Fig. 6b, c). Moreover, four accessions from western China (G1-1) clustered with Middle Asian accessions (G1-2, Fig. 6a), and there was a clear gene flow from the G1-1 group to the G1-2 group (Supplementary Fig. 16), indicating that Middle Asian accessions might have migrated from western China. Besides, the genetic structure of G2 exhibited a gradually changing trend from G2-1 to G2-2. When *K* = 2 and 3, G2-1 possessed both G1-3/G1-1 and G2-2 genetic structures, which illustrated that the phylogenetic relationship between G2-1 and G1 was closer than that between G2-2 and G1(Fig. 6b). In addition, *F*_*ST*_ values between G1 and G2-1 were lower than those between G1 and G2-2 (Fig. 6d). Together with their geographical locations, we speculated that the Japanese, American, and European accessions (G2-2) migrated or were introduced from southeast China (G2-1, Fig. 6e).

### Genomic signals of isoalliin variations in *A. fistulosum*

**Fig. 7:**
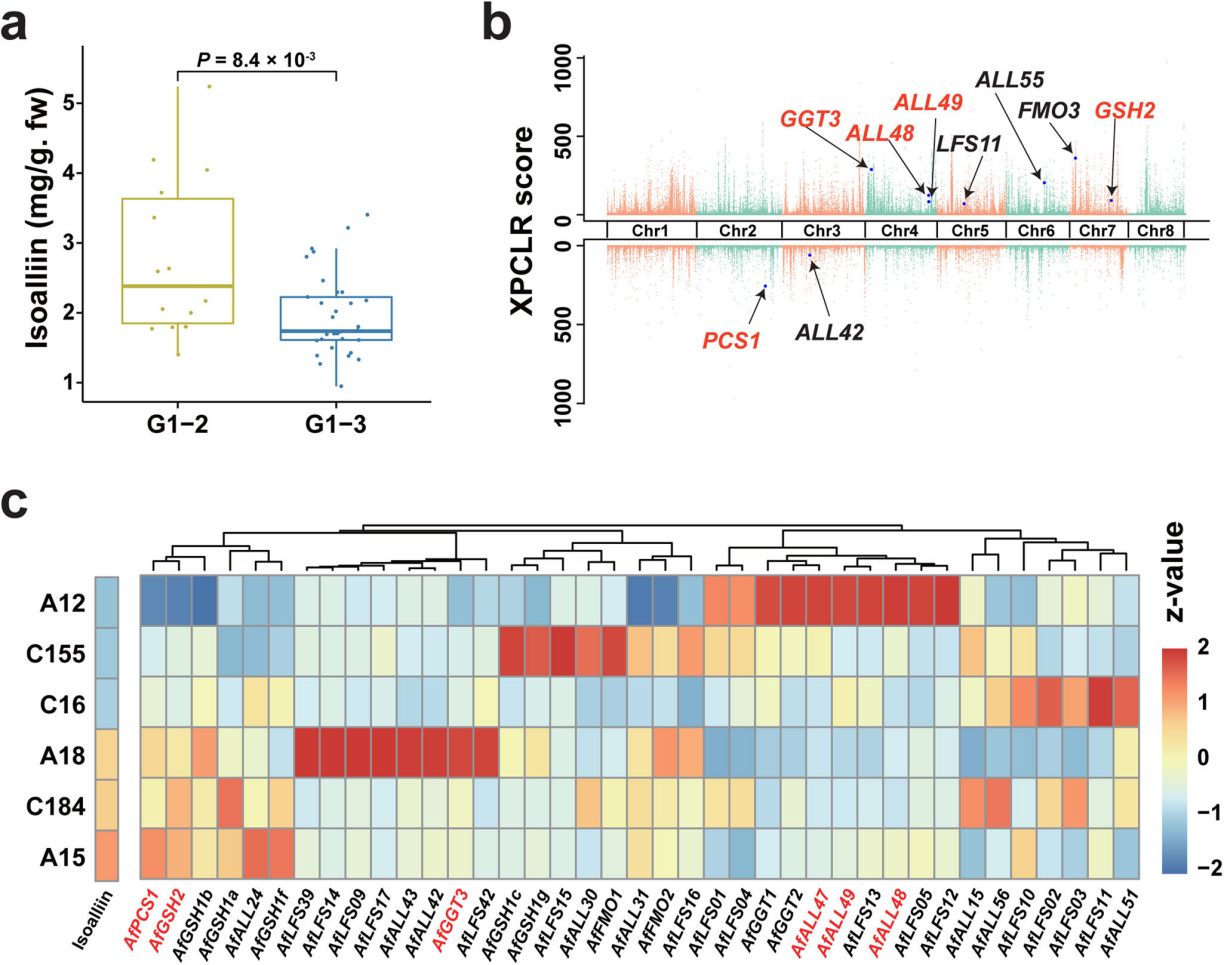
Insights into the variation of isoalliin accumulations in bunching onion accessions. **a** Isoalliin contents in leaves of G1-2 (*n* = 14) and G1-3 (*n* = 33). *P-*values were calculated using the two-tailed Wilcoxon test. In the box plots, the minima, maxima, center bounds of box showed 25%, 75% quartile values and the average values, while the whiskers showed maxima and minima values of isoalliin contents. **b** Top: Genome-wide distribution of selective sweeps in G1-2 compared with G1-3 using XP-CLR (cross-population composite likelihood-ratio test) values. Bottom: Genome-wide distribution of selective sweeps in G1-3 compared with G1-2. **c** Isoalliin concentrations and the gene expression profiles of the sulfur-containing flavor-related genes (those genes with average FPKM < 0.5 was filtered) in the leaves of six accessions. The color scale represents the average isoalliin contents or FPKM Z-value normalized by R software. Source data are provided as a Source Data file.

We quantified the isoalliin contents of 91 *A. fistulosum* accessions to determine the genomic basis of the isoalliin variations. The isoalliin content of G1-2 accessions were observed to be significantly higher than those of G1-3 accessions (Fig. 7a). By comparing the selective sweeps between these two groups, we discovered 2718 and 2133 genes located in the selective regions of G1-2 and G1-3, respectively. Several sulfur-flavor-formation-related genes, such as *AfGSH2*, *AfFMO3*, *AfGGT3*, *AfALL48*, *AfALL49*, *AfALL55*, and *AfLFS11* were under selection in G1-2. *AfPCS1* and *AfALL42* were identified to be under selection in G1-3 (Fig.7b). Moreover, a positive correlation between the endogenous isoalliin amount and expression levels of several ACSO-biosynthesis or -hydrolysis genes was observed in the leaves of six accessions with significant difference in isoalliin content, among which *AfGSH2* and *AfPCS1* exhibited the highest correlation (Fig. 7c). Among the 25 flavor-formation-related genes that were highly expressed in low-isoalliin accessions (A12, C155, and C16), 19 ACSOs hydrolysis-related genes (*ALL* and *LFS*) were identified, implying that both weak biosynthesis and strong hydrolysis may contribute to their lower isoalliin accumulation. Among these 19 ACSOs hydrolysis-related genes, differential *LFS* or *ALL* gene expression patterns in six accessions were observed (Fig. 7c), implying that the extensive expansion of ACSOs hydrolysis-related genes might offer more evolutionary opportunities for the variation of isoalliin contents in bunching onion.

## Discussion

In the present study, we generated a chromosome-level mega-genome of *A. fistulosum*, with a genome size of 11.27 Gb and contig N50 of 7.34 Mb (Table 1). Our reference genome exhibited superior quality based on both major assembly indicators and several assessments (BUSCO, CEGMA, LAI, and Merqury; Supplementary Tables 6 and 7). Undoubtedly, the achievement of this genome assembly will accelerate our understanding of the evolution and relatedness of major *Allium* crops. The genome enlargements of both bunching onion and garlic crops arise from bursts of common *Gypsy*-type LTRs, but they exhibit uneven expansions of the LTR subclades. Meanwhile, DSD events dominated the contributions to gene expansion in major *Allium* crops (Fig. 3). As highly conserved genome synteny has been observed in bunching onion and bulb onion with an SSR-markers based genetic map^21^, high genome collinearity between bulb onion, bunching onion, and garlic was shown at the chromosome level. The chromosomes of the bunching onion (*A. fistulosum*) and bulb onion (*A. cepa*) showed almost one-to-one collinearity (Fig. 2c, Supplementary Fig. 6), indicating high consanguinity between these two *Allium* crops. Therefore, the estimated sequence of each chromosome of the bunching onion can be used as a template for onions with high commonality in the chromosome structure. Meanwhile, this genomic evidence well explains the weak reproductive isolation and ubiquitous existence of interspecific hybridization among *Allium* crops^14–17^, which provides valuable information for directing future genome-aided breeding of *Allium* crops.

The *Allium* genus is characterized by unique flavors and medicinal properties owing to its specific cysteine-derived sulfur compounds^3–5^. We observed that sulfur-metabolism-related genes in GO terms of “Carbon-sulfur lyase activity” and “Cysteine-type peptidase activity” were specifically expanded in the three *Allium* crops examined (Fig. 2b). Importantly, extensive expansion of *ALL* and *LFS* gene families is a striking feature of *Allium* crops. In intact tissues, ACSOs are stored in the cytosol of mesophyll cells. Upon tissue damage, the vacuolar ALL enzyme contacts and hydrolyzes ACSOs to produce sulfenic acids, which are further converted into various sulfur-containing bioactive compounds via spontaneous reactions^5^.Thus, we speculated that the extensive expansion of ACSOs hydrolysis-related genes might lead to the over-accumulation of related transcripts and resultant enzymes, which might be helpful for quick responses to external stimuli. In particular, the LFS gene family was detected only in *Allium* crops among the 14 selected genomes (Fig. 4a). These results strongly support that the *Allium* crops evolved large and special ALL and LFS gene families possibly to resist diseases and insects, by utilizing its volatile and pungent end-products^5–7^.

The species-specific clustering of *ALL* and *LFS* gene families indicated their independent expansions in the three *Allium* crops (Fig. 4, Supplementary Fig. 10), implying that their pungent flavor might have resulted from convergent evolution. It is known that the activity of LFS is reduced to 1/100 or less even by replacing only one amino acid^29^. Therefore, we speculate that the extensive duplication and differentiation of the LFS gene family may lead to the difference of protein activities or functional diversities. LFS genes with high activity and high expression levels are likely involved in the production of tearing properties and characteristic flavors in *Allium* crops. Those members with no or negligible LFS activity, might act in other secondary metabolic pathways instead. It has been reported that different terpene synthase (TPS) subgroups participate in different bioactive terpene biosynthesis^30^. Similarly, *A. fistulosum* and *A. cepa*, preferred to extensively express LFS-c group members, which may function in isoalliin hydrolysis. However, *A. sativum* predominately expresses both LFS-a and LFS-c members (Fig. 4c, d), which might act on alliin and isoalliin decompositions. ACSOs can be decomposed into various sulfur constituents including diallyl and methyl allyl^31^, thus, we conjecture that different clades of the LFS and ALL gene families might play roles in formulating other sulfur-containing bioactive compounds, which need to be further verified in future genetic and biochemical studies.

Moreover, the expansion of ALL and LFS gene families were possibly synchronized with the proliferation of LTRs (Supplementary Fig. 11)^23, 24^. In addition, *FMO* genes, involved in the biosynthesis of both ACSOs and glucosinolates, underwent extensive expansions only in *Arabidopsis* among the 14 selected genomes (Fig. 4a), which might be related to the accumulation of sulfur-containing glucosinolates in *Cruciferous* crops^32, 33^. However, the absence of several glucosinolate-related genes in *Allium* crops suggested that they might select different evolutionary directions toward the biosynthesis of sulfur-containing bioactive compounds, compared with *Cruciferous* plants (Fig. 4a and Supplementary Fig. 17).

Traditional *Allium* categories cause confusion in evolutionary studies and exploitation of agricultural usage, mainly because of the lack of genome information. Previously, *A. altaicum* was commonly considered the ancestor of *A. fistulosum* based on its phenotypic resemblance and noncoding chloroplast DNA similarity^12, 34^ (Supplementary Fig. 12). Here, we found that it did not cluster with *A. fistulosum* in either SNP-based phylogeny or PCA analyses, but was phylogenetically closer to the Chinese red onion (Fig. 6a and Supplementary Data 4). Thus, we postulated that *A. altaicum* is closely related to *A. fistulosum*, rather than its proposed ancestor. In contrast to earlier morphological classifications, three storey onions (*A. fistulosum* var*. viviparum*) accessions and the Chinese red onions (*A. cepa* var*. proliferum*), propagated by aerial bulbs, were clustered into two different groups (Fig. 6a, Supplementary Fig. S18 and Supplementary Data 4). Their characterized aerial bulbs, which could be used for asexual propagation, may have resulted from convergent evolution.

The geographic positions of G1-1 are close to the major diversity center (ranging from the Mediterranean Basin to Central Asia) for *Allium* genus^2^, and the highest nucleotide diversity was observed in this subgroup (Fig. 6d). These findings are consistent with the historical records that northwestern China was the origin center of *A. fistulosum*^35, 36^. In addition, the literature on the cultivation of *A. fistulosum* in China dates back to the third century BC. The Japanese *A. fistulosum* accessions were considered to be introduced from China at approximately 720 AC. Thereafter, *A. fistulosum* was spread to Western Europe during or at the end of the Middle Ages (1000-1400 AC) from Asia^35–37^. These records are supportive of our phylogenetic results that the G2-2 (accessions from Japan and America and European countries) might have migrated from southern China.

Furthermore, among *A. fistulosum*, diverse accessions exhibited distinct geographic clustering rather than morphological classification in phylogenomic analysis (Fig. 6b). Middle Asian accessions (G1-2), which are distributed in Siberia and its adjacent areas, might migrate from western China (G1-1), whereas Japanese, American, and European accessions (G2-2) migrated or were introduced from southeast China G2-1 (Fig. 6e). Collectively, we validate that Northwestern China (G1-1) is the single origin center of *A. fistulosum*.

In summary, the high-quality chromosome-level genome of *A. fistulosum* greatly advances our understanding of genome diversity and expansion of *Allium* crops. The highest diversification of China-originated accessions and the existence of close relatives indicate that China is the primary origin and domestication center for *A. fistulosum* crops. The expansion and differentiation of the alliinase and lachrymatory factor synthase gene families might relate to *Allium*-specific flavor formation. Our current study will enable further functional genomic studies and genomic selection of important agronomic traits in *Allium* crops.

## Methods

### Plant materials and sequencing libraries preparations

The bunching onion accession “SXSJC” (grown in Hangzhou, Zhejiang Province, China) was sequenced for genome assembly. Detailed information on the 135 *Allium* accessions used for re-sequencing is provided in Supplementary Data 4. A total of 101 *A. fistulosum* accessions were distributed worldwide, including in China, Japan, and European, American, and Central Asian countries. Shallots and Chinese red onions were collected from China, and the feral *A. altaicum*, which was considered the closest relative and potential ancestor for cultivated *A. fistulosum*^13, 38^, was collected from the Altai Mountains (89.44E, 47.05 N; Xinjiang, China).

For genome assembly, genomic DNA was extracted from the young leaves of bunching onions using a DNAsecure Plant Kit (Tiangen Biotech, Beijing, China), and DNA quality was detected by agarose gel electrophoresis. Single molecular real-time (SMRT) PacBio sequencing libraries were built following the standard protocols of Pacific Biosciences. Briefly, high-quality genomic DNA was sheared to ~20 kb, the damaged ends were repaired and ligated with a blunt-end adaptor, and the resulting libraries were sequenced using the PacBio Sequel platform.

For Bionano sequencing, DNA was extracted using the suggested kits (Bionano Prep Plant DNA Isolation Kit), and the specific sequences across the entire genome were labeled. The labeled DNA was transferred into a cartridge for scanning, and the images were converted into molecules.

Hi-C libraries were constructed according to the suggested procedure. Briefly, leaf samples were fixed with formaldehyde solution before chromatin extraction, and chromatin was digested with 400U of DPNII restriction enzyme at 37 °C. DNA ends were labeled with biotin, and DNA ligation was performed using T4 DNA ligase (NEB). After ligation, proteinase K was added for reverse crosslinking. The DNA fragments were then purified and dissolved. The purified DNA was fragmented to 300–500 bp, and the DNA ends were repaired. Biotin-labeled DNA fragments were separated using Dynabeads® M-280 Streptavidin (Life Technologies). Hi-C libraries were controlled for quality and sequenced on an Illumina HiSeq X Ten sequencer.

For Illumina sequencing, DNA libraries were constructed according to a previously described procedure (Illumina). Briefly, DNA was broken randomly into segments using a Covaris ultrasonic crusher, DNA ends were repaired, and poly (A) adaptors were added. PCR was performed using purified DNA, and the final libraries were sequenced on an Illumina platform after assessment.

### Genome assembly and quality assessment

The genome size, heterozygosity ratio, and repeat sequence ratio were evaluated by *k-mer*^39^ distribution analysis (k = 17) using Illumina short-reads. PacBio reads were used to assemble the contig-level genome using CANU^40^ (v1.9, parameters: genomeSize = 12 G, corOutCoverge = 40 G). The contigs were polished with PacBio reads using NextPolish (v1.2.4; rerun = 3, https://github.com/Nextomics/NextPolish) and Illumina short-reads using Pilon (v1.2.2)^41^. Subsequently, redundant sequences were removed using Purge_dups (v1.2.3)^42^. Bionano data were used for auxiliary assembly using Bionano Solve (v3.5.1, DeNovo Assembly: -i 5 -F 1 -W 1 -c 1, Hybrid Scaffold: -B 2 -N 2) to improve the accuracy of the assembly^43^. Hi-C data were used to assemble the chromosome-level genome using ALLHiC (v0.9.8, https://github.com/tangerzhang/ALLHiC) software.

Complementary methods were employed to evaluate the quality of the genome assembly. First, genome completeness was assessed based on conserved plant genes in the BUSCO (v3.0.2) and CEGMA (v2.5) databases. Second, Illumina short-reads were mapped to the assembled genome using BWA (v0.7.8)^44^ to assess coverage rate and average depth. The LTR_retriever (v1.0.7)^22^ package was used to evaluate genome quality. Mequery (v1.3) was used to assess the consensus quality (QV) value and completeness of the genome assembly^20^. The markers of genetic map^21^ were mapped to our genome using Chromonomer (v1.07, https://github.com/jleluyer/chromonomer_workflow), and collinear relationships were displayed using Python scripts.

### Genome annotations

RepeatMasker and RepeatProteinMask (v4.07)^45^ were used to identify TEs by alignment to the repeat library (Repbase v15.02), whereas de novo prediction of TEs was performed using RepeatModeler (v1.05, http://www.repeatmasker.org/RepeatModeler/, version 1.0.5), RepeatScout, and LTR_FINDER. Tandem Repeats Finder (TRF, v4.09) analysis was performed to identify the tandem repeats in the genome.

De novo prediction was performed using five ab initio gene prediction programs: Augustus (v3.2.3)^46^, Geneid (v1.4, https://genome.crg.cat/software/geneid/index.html), Genescan (v1.0, http://genes.mit.edu/GENSCAN.html), GlimmerHMM (v3.0.2, https://ccb.jhu.edu/software/glimmerhmm/), and SNAP (v2013.11.29, https://snap.stanford.edu/). The protein sequences of 12 species (*A. sativum*^11^*, A. cepa*^25^, *Asparagus setaceus*^47^*, Asparagus officinalis*^48^*, Dendrobium officinale*^49^*, Apostasia shenzhenica*^50^*, Phalaenopsis equestris*^51^, *Phoenix dactylifera*^52^*, Oryza sativa*^53^, *Ananas comosus*^54^*, Amborella trichopoda*^55^, *and Musa schizocarpa*^56^) from NCBI or ENSEMBLE were aligned against the *A. fistulosum* genome using TBLASTN (v2.2.26, E-value <10e-5), which was used to predict gene models. Tophat (v2.0.13, http://ccb.jhu.edu/software/tophat/index.shtml) and Cufflinks (v2.1.1, http://cufflinks.cbcb.umd.edu/) pipelines were used to map the RNA-seq data to the *A. fistulosum* genome for gene prediction. Trinity (v2.1.1) and PASA (v2.2.0)^57^ were used to analyze the gene structures. All gene models predicted using these approaches were merged using the weighted and non-redundant gene set 1.1.1 (EVM, v1.1.1, http://evidencemodeler.github.io/).

Gene annotation was performed by blasting the SwissProt, TrEMBL, and NCBI non-redundant protein databases, and the best hits were selected for annotations. Motifs and domains were annotated using InterProScan (v4.7) to search the InterPro (v29.0) databases. The GO and KEGG pathways for each gene were obtained using the best-match classification. Noncoding RNAs were predicted using the tRNAscan-SE (v1.4)^58^ and INFERNAL (v1.1.2)^59^ software.

### Analysis of WGD events

FASTKs (https://github.com/mrmckain/FASTKs) was used to calculate the synonymous substitution (*Ks*) values of the paralogous blocks in *A. fistulosum*. The density estimation of the *Ks* value distributions was based on finite normal mixture modeling using MCLUST (https://sites.stat.washington.edu/mclust/), and the distribution of Ks values was used to determine WGD events.

### Comparative genomics and genome evolution analysis

Briefly, orthologous gene families (single-copy and multi-copy families) of *A. fistulosum* and other genomes (*A. sativum*^11^, *A. cepa*^25^, *Asparagus setaceus*^47^, *Asparagus officinalis*^48^, *Dendrobium officinale*^49^, *Apostasia shenzhenica*^50^, *Phalaenopsis equestris*^51^, *Phoenix dactylifera*^52^, *Oryza sativa*^53^, *Ananas comosus*^54^, *Amborella trichopoda*^55^ and *Musa schizocarpa*^56^) were obtained using OrthoMCL (v1.4, http://orthomcl.org/orthomcl/). A super-alignment matrix was obtained by aligning single-copy gene families with MUSCLE (v3.8.31, http://www.drive5.com/muscle/), which was subsequently used for ML phylogenetic tree construction using RaxML (ML tree, Model GTRGAMMA, v8.2.12, http://sco.h-its.org/exelixis/web/software/raxml/index.html). Mcmctree (burn-in = 10,000, sample number = 100,000, sample frequency = 2; http://abacus.gene.ucl.ac.uk/software/paml.html) was used to calculate the divergence time based on the TimeTree database (http://www.timetree.org/) and a previous report^55^. Gene family expansion and contraction were detected using CAFÉ (v4.2)^60^ software. Genomic collinearity between garlic and bunching onions was analyzed using the MCScanx pipeline^61^.

### Insertion time and phylogenetic analysis of LTRs

The ends of the LTR retrotransposons were aligned using MUSCLE (v3.8.31, (http://www.drive5.com/muscle/), and the insertion time (T) of the LTRs was calculated using the formula T = K/2r. K is the genetic distance calculated using the formula K = −0.75ln (1-4λ/3). The nucleotide divergence rate (λ) between the two LTRs less than 0.75 was retained for further analysis. The nucleotide substitution rate “r” was set to 1.3e-8 substitutions per site per year. Uncorrected pairwise distances were used to construct a neighbor-joining unrooted phylogenetic tree using TreeBest (v1.9.2, http://treesoft.sourceforge.net/treebest.shtml) with the suggested parameters. We classified LTRs into Copia, Gypsy, and other superfamilies using LTRdigest (v1.07)^62^, while the secondary LTRs were determined according to their phylogenetic properties.

### Identification of ancestral and new DSD genes

Gene duplication events were classified as dispersed, proximal, WGD, tandem, and transposed duplications using the DupGen_finder (https://github.com/DXXDR/DupGen_finder) pipeline. We investigated the global landscape of gene duplications in 13 selected genomes and several large genomes, including three large genomes of *Ginkgo biloba*^63^, rye^64^, and Chinese pine^65^. Chromosome collinearity was analyzed to determine whether a gene was located in the collinearity region based on JCVI^61^. Both *A. sativum* and *A. cepa* used *A. fistulosum* as the reference genome, whereas *A. sativum* was selected as the reference genome for *A. fistulosum*. Genes in collinear regions were considered ancestral DSD genes, whereas those outside the collinear regions were considered new DSD genes. We selected dispersed gene pairs in which one was an ancestral gene and the corresponding gene was a new gene to analyze the expression difference.

### Identification and evolution analysis of flavor-related genes

To identify genes involved in the sulfur-metabolism biosynthesis pathways, the key parameters of BLASTP (v 2.2.26) were E-value <1e-5 and the identity >50%. The protein domains of the candidate genes were identified using HMMER (v3.1b1) software, and genes with different domains were filtered. The protein sequence of bulb onion LFS gene (GenBank accession no. AB089203) and garlic ALL genes (GenBank accession no. AAB32477.1, ACN78838.1, AAD26853.1, and AAA32639.1) from the NCBI database were used to identify the candidate orthologs. The query sequences of glucosinolate-related genes were obtained from a review^33^. Full-length amino acid sequences of LFS and ALL genes were aligned using MUSCLE (v3.8.31), and a phylogenetic tree was constructed using Treebest (v1.9.2, http://treesoft.sourceforge.net/treebest.shtml) and ITOLs (https://itol.embl.de/).

### Spatial expressions of the sulfoxide-related genes

Illumina RNA-seq was performed on the roots, pseudo-stems, leaves, floral stalks, and flowers of bunching onions, and the roots, bulbs, leaves, and pseudo-stems of bulb onions with four biological replicates. Gene expression datasets were obtained using the Tophat (http://ccb.jhu.edu/software/tophat/index.shtml) and Cufflinks (http://cufflinks.cbcb.umd.edu/) pipelines. The expression patterns of the target genes were visualized using a heatmap library (https://CRAN.R-project.org/package=pheatmap) in the R software with log_2_ (FPKM + 1) values.

### Re-sequencing and population genomics analysis

A total of 350 bp pair-end reads from 135 accessions were generated using the Illumina HIseq2500 platform. The mapping rates of *A. ascalonicum* and *A. cepa* var*. proliferum* were less than 90, and 4 × genomic coverage was less than 60%. We used 96 *A. fistulosum* accessions to filter SNPs. The SNPs were subjected to a quality control procedure following these steps: (1) removing the Illumina library construction adapters, (2) removing the reads containing more than 10% unknown bases (N bases), and (3) removing the low-quality reads containing more than 50% of low-quality bases (sequencing quality value ≤ 5). BWA (v0.7.8)^44^ was used to align the clean reads to the reference genome, and Samtools (https://github.com/samtools/samtools) was used to call SNPs/InDels with the following parameters (-q 1 -C 50 -m 2 -F 0.002 -d 1000). The raw SNP/InDel sets with a mapping quality <20, or a depth of the variant position <3, or a missing rate >0.05, or an SNP frequency <5% were filtered. After filtering, we selected high-quality SNPs from the 135 *Allium* accessions for subsequent analyses. ANNOVAR (v20191024, https://annovar.openbioinformatics.org/en/latest/) was used for the functional annotation of variants. A phylogenetic tree was constructed using SNPhylo (v20180901)^66^. The population structure of the accessions was investigated using Admixture (v1.23). In addition, PCA was performed using VCFtools (v0.1.12b, https://vcftools.github.io/index.html). Linkage disequilibrium analysis (LD decay) was calculated using PopLDdecay (v03.41)^67^. The XP-CLR scores were calculated using XP-CLR (v1.0) with a sliding window size of 40 kb and a step size of 20 kb^68^.The top 5% region was selected as the candidate selection region.

### Determination of ACSOs in *Allium* crops

To prevent ACSO degradation, O-(carboxymethyl) hydroxylamine hemihydrochloride (OCMHA) was used to inhibit the activity of alliinase^69^. The extraction procedure was as follows: 0.15–0.20 g flesh weight samples were transferred to 2 mL centrifuge tube containing a 5 mm steel ball, and 990 μL OCMHA solution (1.1 g/L methanol/deionized water 4:1, v/v) and 10 μL L-norleucin solution (1 mg/mL water) were added. After rubbing with a tissue grinding machine, the samples were homogenized for 1 min. The resulting slurry was centrifuged (10 min, room temperature, 12,000 rpm) and the supernatant was then diluted 10,000 × *g*. with a mixture of acetonitrile and deionized water (4:1, v/v). The final extracts were stored in vials at −20 °C until analysis.

ACSOs were identified by comparison with the authentic standards (alliin, Beijing SoIarbio Life Science Company, Beijing, China; isoalliin, from Chengdu Caoyuankang Biotechnology Company, Chengdu, China; and methiin, from Shanghai Yuanye Biotechnology Company, Shanghai, China). Mixed calibration curves were used at concentrations from 0.01 to 500 ng/mL for ACSOs quantification. The ACSO content was determined by Agilent 1290 ultra-high-performance liquid chromatographer coupled to a 6470 triple quadrupole spectrometer (Agilent Technologies, Waldbronn, Germany). The chromatographic separation was performed on a Waters BEH amide column (100 × 2.1 mm, 1.7 μm) maintained at 60 °C with a flow rate of 0.6 mL/min. The mobile phase consisted of (A) water with 0.5% formic acid and (B) acetonitrile with 0.5% formic acid. A gradient elution was used: 0–4 min, 10–15% A; 4–8 min, 15–60% A; 8–15 min, 10% A; injection volume 1 μL. Ionization was conducted in positive ion electrospray mode, and the operating parameters were as follows: capillary voltage, 3000 V; drying gas temperature, 325 °C; drying gas flow, 5 L/min; nebulizer pressure, 45 psi; sheath gas temperature, 350 °C; and sheath gas flow, 11 L/min. In addition, three biological replicates were used for all the accessions.

### Reporting summary

Further information on research design is available in the Nature Portfolio Reporting Summary linked to this article.

## Supplementary information

Supplementary information

Description of Additional Supplementary Files

Supplementary Data 1

Supplementary Data 2

Supplementary Data 3

Supplementary Data 4

Reporting Summary

## Data Availability

All raw sequencing data generated in this study have been deposited to CNSA (https://db.cngb.org/cnsa/) under accession CNP0002276. The expression data of *A. sativum* were downloaded from NCBI GEO database under accession GSE145455. Source data are provided with this paper.

## Source data

Source Data
