# Supplementary material for: Chromosome-level genome assembly of bunching onion illuminates genome evolution and flavor formation in Allium crops
Source: Nat Commun. 2022 Nov 5;13:6690. doi: 10.1038/s41467-022-34491-3 (PMC9637129; doi:10.1038/s41467-022-34491-3)
Supplement: Supplementary file 1 — Supplementary information [file 41467_2022_34491_MOESM1_ESM.pdf]

Chromosome-level genome assembly of bunching onion illuminates genome evolution and flavor formation in *Allium* crops

Liao *et al.*

Supplementary Table 1. Summary of the sequencing data for *A. fistulosum* genome assembly.

Libraries	Insert size	Total data (G)	Read length (bp)	Sequence coverage (X)
**Illumina reads**	350bp	1389.89	150	116.29
**PacBio reads**	--	1649.82	--	138.04
**Bionano**	--	1020.22	--	85.36
**HiC**	--	1136.41	--	95.08
**Total**	--	5196.34	--	434.77

Supplementary Table 2. Estimated genome statistics using *k-mer* analysis (*k* = 17).

Depth	Number of *k-mer*	Genome size(M)	Revised genome size(M)	Heterozygous rate(%)	Repeat rate(%)
87	1,041,421,564,963	11,970.36	11,951.73	0.64	89.89

Supplementary Table 3. Summary of the genome assembles for *A. fistulosum*.

	Pacbio+illumina+illumina-pilish+pacbio_polish	Pacbio+illumina+illumina-pilish+pacbio_polish+bionano		Pacbio+illumina+illumina-pilish+pacbio_polish+bionano+hic	
	**contig**	**contig**	**scaffold**	**contig**	**scaffold**
**Total_length**	11,273,877,593	11,273,877,473	11,293,432,26	11,273,877,473	11,293,897,766
**Total_number**	7,619	8,126	7,352	8,278	2,855
**Max_length**	87,932,624	83,135,306	90,340,743	83,135,306	1,725,086,671
**N50**	8,417,756	7,415,081	8,980,620	7,345,229	1,386,005,700
**N60**	6,233,651	5,235,816	6,549,398	5,176,956	1,327,381,468
**N70**	4,302,056	3,625,928	4,427,297	3,565,919	1,223,795,258
**N80**	4,302,056	2,221,495	2,740,631	2,197,287	1,131,948,510
**N90**	4,302,056	1,061,145	1,307,006	1,055,328	1,109,041,757

Supplementary Table 4. Summary of the genome characteristics of assemble using HiC data.

Sequeues ID	Cluster number	Sequeues length
**Chr1**	654	1,641,299,136
**Chr2**	713	1,725,086,671
**Chr3**	553	1,223,795,258
**Chr4**	555	1,109,041,757
**Chr5**	470	1,131,948,510
**Chr6**	603	1,386,005,700
**Chr7**	570	1,603,727,048
**Chr8**	545	1,327,381,468

Supplementary Table 5. Summary of Hi-C mapping.

Class	Scaffold number	Total length	Percentage of the genome
**Placed**	8	11,148,285,548	98.71%
**Unplaced**	2,847	145,612,218	1.29%
**Total**	2,855	11,293,897,766	100%

Supplementary Table 6. Assembly assessments by BUSCO.

	C	S	D	F	M
**Pacbio+illumina+illumina-pilish+pacbio_polish**	91.90%	84.00%	7.90%	2.30%	5.80%
**Pacbio+illumina+illumina-pilish+pacbio_polish+bionano**	91.70%	84.00%	7.70%	2.50%	5.80%
**Pacbio+illumina+illumina-pilish+pacbio_polish+bionano+hic**	91.00%	88.00%	3.00%	2.90%	6.10%
**Annotated_genes**	93.80%	84.10%	9.70%	3.20%	3.00%

#C: Complete Single-Copy BUSCOs; S：Complete and single-copy BUSCOs; D: Complete Duplicated BUSCOs; F: Fragmented BUSCOs; M: Missing BUSCOs; n: Total BUSCO groups searched

Supplementary Table 7. Assembly assessments using GEGMA.

cies	Complete		Complete + Partial	
	# Prots	%Completeness	# Prots	%Completeness
**Pacbio+illumina+illumina-pilish+pacbio_polish**	221	89.11	232	93.55
**Pacbio+illumina+illumina-pilish+pacbio_polish+bionano**	220	88.71	231	93.15
**Pacbio+illumina+illumina-pilish+pacbio_polish+bionano+hic**	222	89.52	234	94.35

Supplementary Table 8. Assembly assessments by Illumina read mapping.

Version	Mapping rate (%)	Average sequencing depth	Coverage (%)	Coverage at least 4X (%)	Coverage at least 10X (%)	Coverage at least 20X (%)	SNP percentage (%)	Heterozygosis SNP (%)	Homology SNP (%)
**Pacbio+illumina+illumina-pilish +pacbio_polish**	99.65	95.48	99.34	99.26	99.17	99.06	0.2222	0.2213	0.0009
**Pacbio+illumina+illumina-pilish +pacbio_polish+bionano**	99.65	95.48	99.34	99.26	99.17	99.06	0.1912	0.190571	0.0629
**Pacbio+illumina+illumina-pilish +pacbio_polish+bionano+hic**	99.65	95.09	99.34	99.25	99.17	99.06	0.0064	0.0063	0.0001

Supplementary Table 9. Summary of repeated sequence identifications.

Type	Repeat size (bp)	Percentgage of genome (%)
**TRF**	540,922,313	4.79
**Repeatmasker**	7,409,524,033	65.61
**Proteinmask**	1,932,359,143	17.11
**Total**	7,884,598,942	69.81

Supplementary Table 10. Summary of repeated sequence annotations.

	De novo +Repbase length(bp)	Percentgage of genome (%)	TE proteins length(bp)	Percentgage of genome (%)	Combined TEs length(bp)	Percentgage of genome (%)
**DNA**	333,089,856	3	109,476,349	1	414,256,264	4
**LINE**	122,505,505	1	142,173,362	1	235,142,630	2
**SINE**	715,356	0	0	0	715,356	0
**LTR**	6,830,517,902	60	1,684,737,702	15	7,021,961,730	62
**Unknown**	42,374,783	0	0	0	42,374,783	0
**Total**	7,409,524,033	66	1,932,359,143	17	7,747,379,863	69

Supplementary Table 11. Summary of gene prediction of *A. fistulosum*.

	Gene set	Number	Average transcript length (bp)	Average CDS length (bp)	Average exons per gene	Average exon length (bp)	Average intron length (bp)
**De novo**	Augustus	272,843	3,004.71	555.83	2.42	229.7	1,724.76
	GlimmerHMM	671,869	15,283.96	326.89	2.72	120.32	8,712.55
	SNAP	546,223	27,726.64	562.93	4.92	114.43	6,930.70
	Geneid	695,655	3,971.93	358.51	2.6	137.72	2,253.83
	Genscan	472,751	13,987.22	589.45	3.97	148.54	4,513.72
**Homolog**	*Allium_sativum*	303,554	1,271.83	550.69	1.85	297.33	846.3
	Ananas_comosus	182,490	1,449.00	639.29	1.93	331.92	874.38
	Apostasia_shenzhenica	351,899	1,874.93	1,119.76	1.73	647.24	1,034.38
	Arabidopsis_thaliana	187,355	1,209.95	600.08	1.68	357.53	898.98
	Musa_schizocarpa	371,952	1,040.64	558.86	1.6	348.29	796.9
	Oryza_sativa	286,142	1,582.11	920.34	1.65	557.25	1,015.66
	Zea_mays	172,758	2,096.13	1,131.14	2.02	560.96	949.39
**RNAseq**	PASA	83,977	5,702.02	892.38	4.29	207.92	1,461.01
	Cufflinks	105,751	8,228.11	1,834.73	4.5	407.39	1,824.81
**EVM**		309,634	2,813.85	548.57	2.37	231.34	1,651.94
**Pasa-update***		309,388	2,818.94	548.81	2.37	231.75	1,659.27
**Final set***		62,255	5,000.13	820.11	3.93	208.47	1,424.73

Supplementary Table 12. Characterization of genes of *A. fistulosum.*

Species	Number	Average transcript length (bp)	Average CDS length (bp)	Average exons per gene	Average exon length (bp)	Average intron length (bp)
Allium_fistulosum	62,255	5,000.13	820.11	3.93	208.47	1,424.73
Allium_sativum	57,561	5,202.81	797.13	3.64	218.96	1,668.52
Musa_schizocarpa	32,809	4,040.61	1,126.84	5.08	222.02	714.97
Ananas_comosus	27,024	4,341.80	1,171.28	5.53	211.68	699.41
Arabidopsis_thaliana	27,302	1,888.02	1,230.07	5.16	238.38	158.15
Apostasia_shenzhenica	21,743	6,147.71	1,099.28	4.5	244.13	1,441.26
Zea_mays	39,188	3,472.15	1,179.62	4.93	239.33	583.5
Oryza_sativa	42,355	2,198.36	992.95	3.9	254.47	415.36

Supplementary Table 13. Summary of gene annotations using different databases.

	Number	Percent (%)
**Total**	62,255	-
**Swissprot**	38,081	61.2
**Nr**	50,431	81
**KEGG**	37,702	60.6
**InterPro**	60,484	97.2
**GO**	53,819	86.4
**Pfam**	36,406	58.5
**Annotated**	61,218	98.3
**Unannotated**	1,037	1.7

Supplementary Table 14. Summary of non-coding RNA predication.

Type		Copy (w*)	Average length (bp)	Total length (bp)	Percentgage of genome (%)
**miRNA**		1,361	106.02	144,289	0.001278
**tRNA**		4,365	74.58	325,561	0.002883
**rRNA**	rRNA	1,416	186.77	264,462	0.002342
	18S	219	581.37	127,319	0.001127
	28S	219	138.71	30,378	0.000269
	5.8S	94	96.54	9,075	0.00008
	5S	884	110.51	97,690	0.000865
**snRNA**	snRNA	1,723	114.33	196,988	0.001744
	CD-box	992	105.95	105,107	0.000931
	HACA-box	203	134.83	27,370	0.000242
	splicing	526	121.81	64,073	0.000567

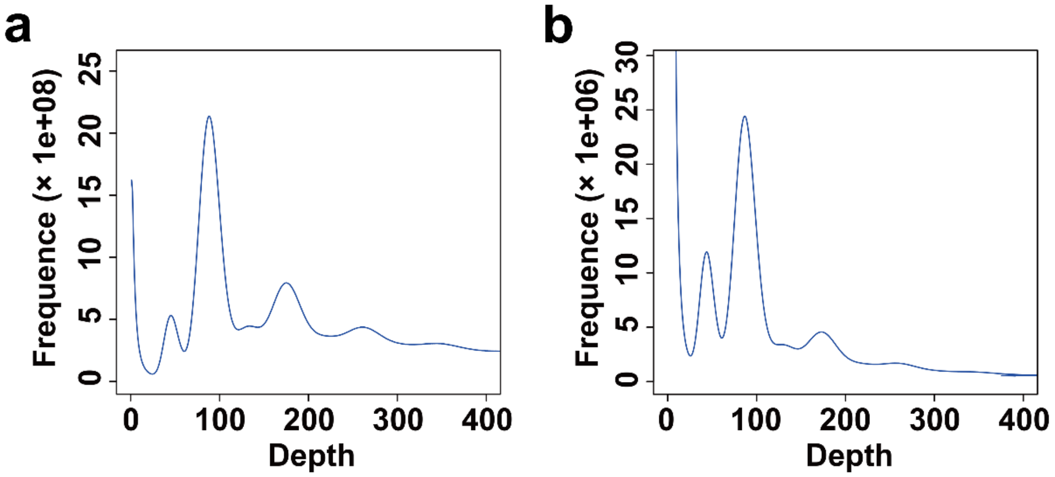

Supplementary Fig. 1. *k-mer* based genome survey of *A. fistulosum*. The frequency plots of *k-mer* number (**a**) and *k-mer* types (**b**), as well as depth given that *k-mer* (k=17).

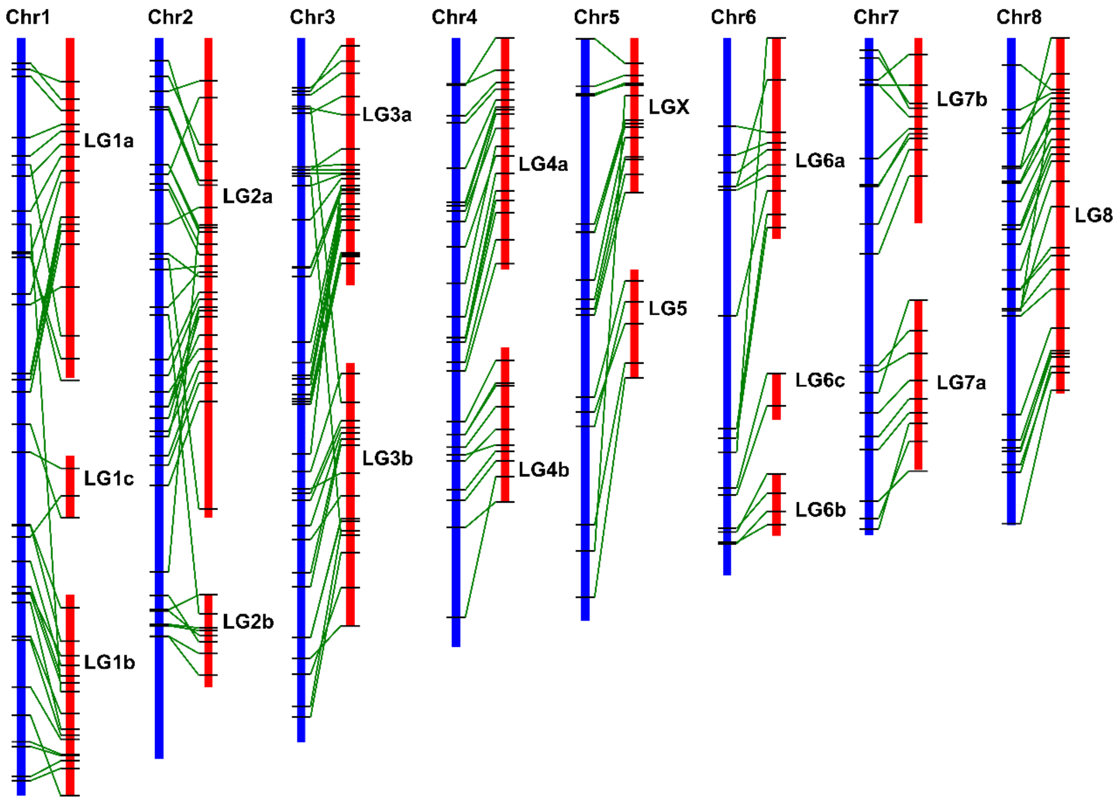

Supplementary Fig. 2. The collinearity between genetic map and *A. fistulosum* genome assembly. The blue columns indicated the chromosomes of assembled genomes, while the red columns indicated linkage groups (LG) of genetic map [21] and green lines indicated the collinearity relationships by SSR-markers anchoring.

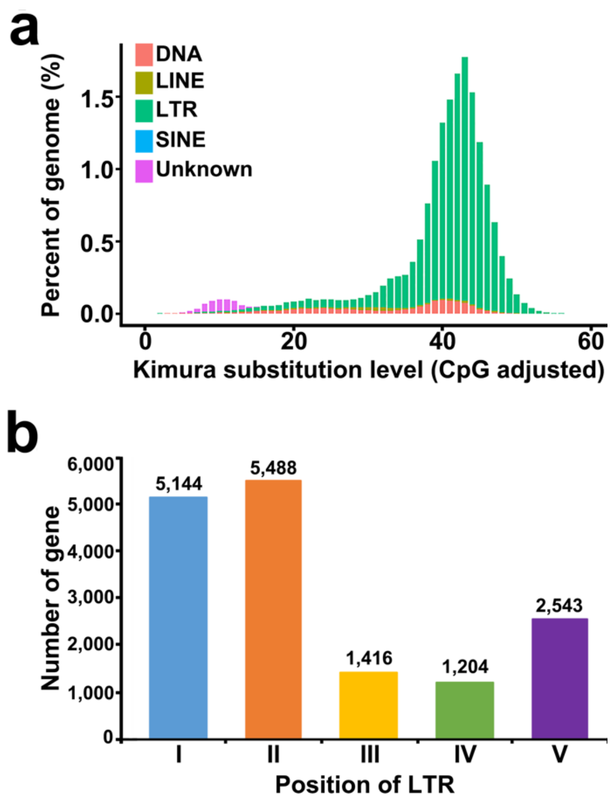

Supplementary Fig. 3. LTR contributions to the genome enlargement of *A. fistulosum*. a, Kimura substitution level (%) for each copy against its consensus sequence used as proxy for expansion history of the transposable elements. LINE, long interspersed nuclear elements; LTR, long terminal repeat; SINE, short interspersed nuclear element. **b,** Numbers of genes inserted by LTR retrotransposons. Blue column (I) indicates the number of genes whose promoter region was inserted by LTR; Orange column (II) indicates the number of genes whose 3'-terminus region was inserted by LTR; Yellow column (III) indicates the number of genes whose encoding region was inserted by LTR; Green column (IV) shows the number of genes that located within the LTR, and purple column (V) indicates the number of genes with sequences that partially overlap with the LTR.

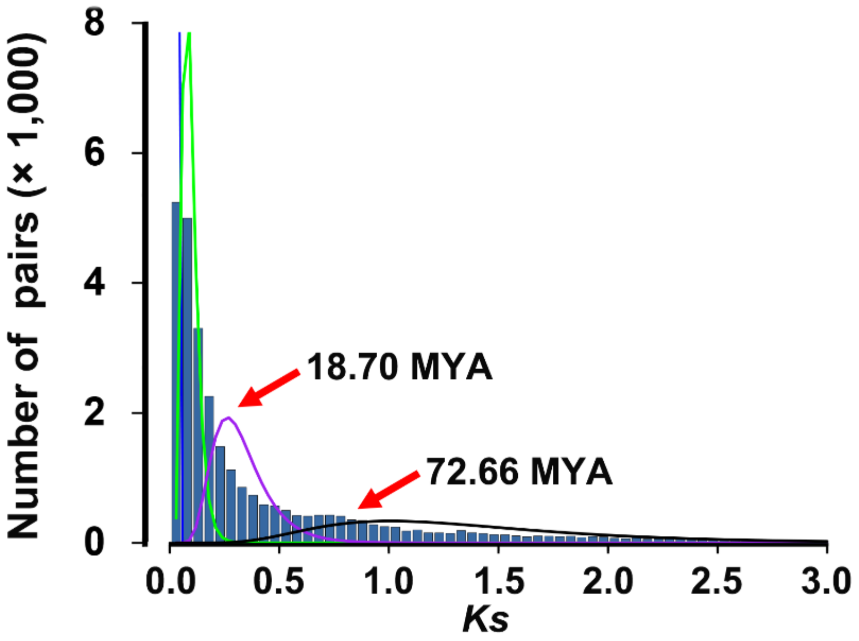

Supplementary Fig. 4. WGD events estimated from the *Ks* value of homologues pairs in syntenic regions of *A. fistulosum* and *A. stavium*. The histplot (blue bars) exhibited the distribution of the *Ks* values and the lines indicated the density estimation of the *Ks* values distributions.

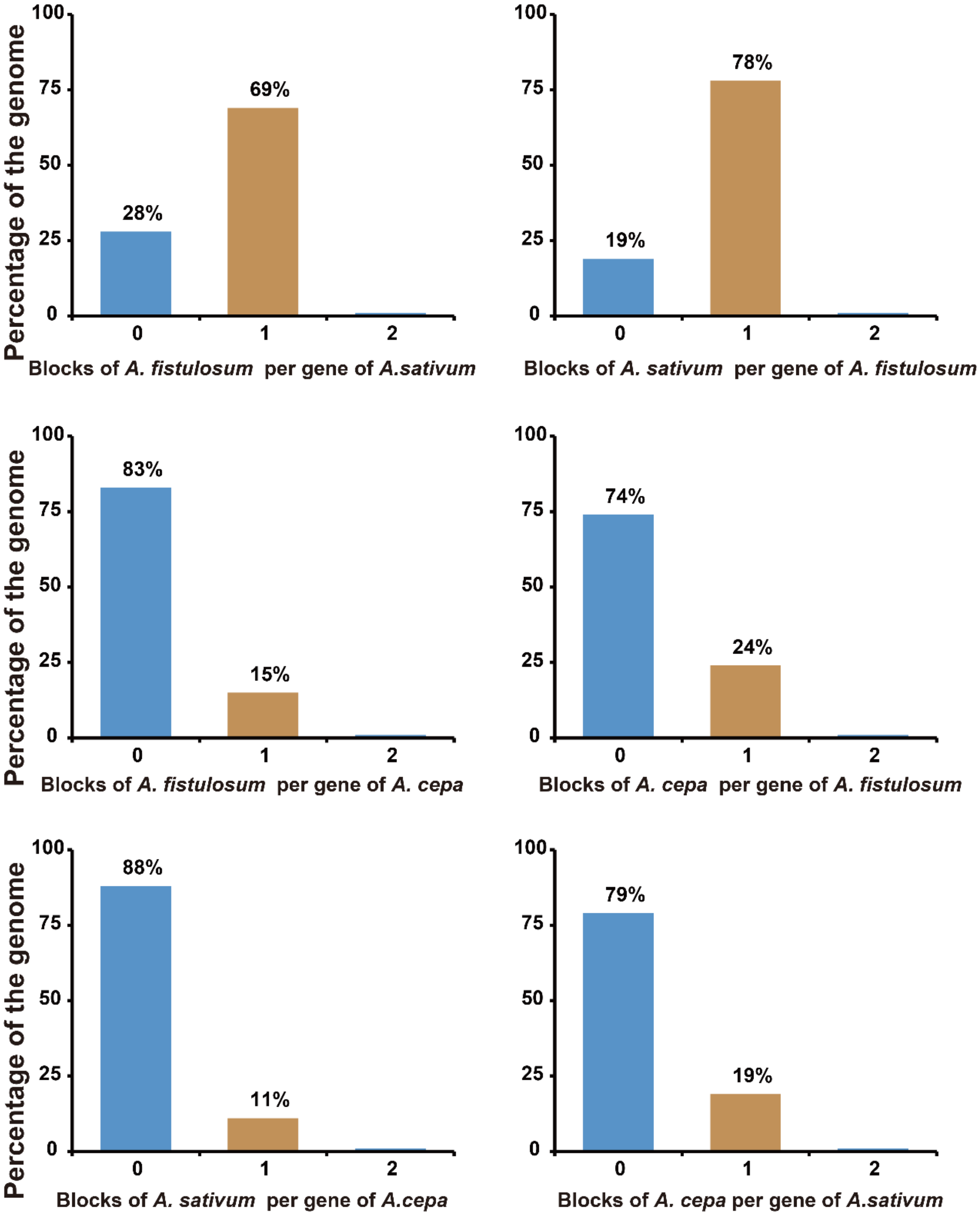

Supplementary Fig. 5. Distribution of blocks of three *Allium* crops against each other.

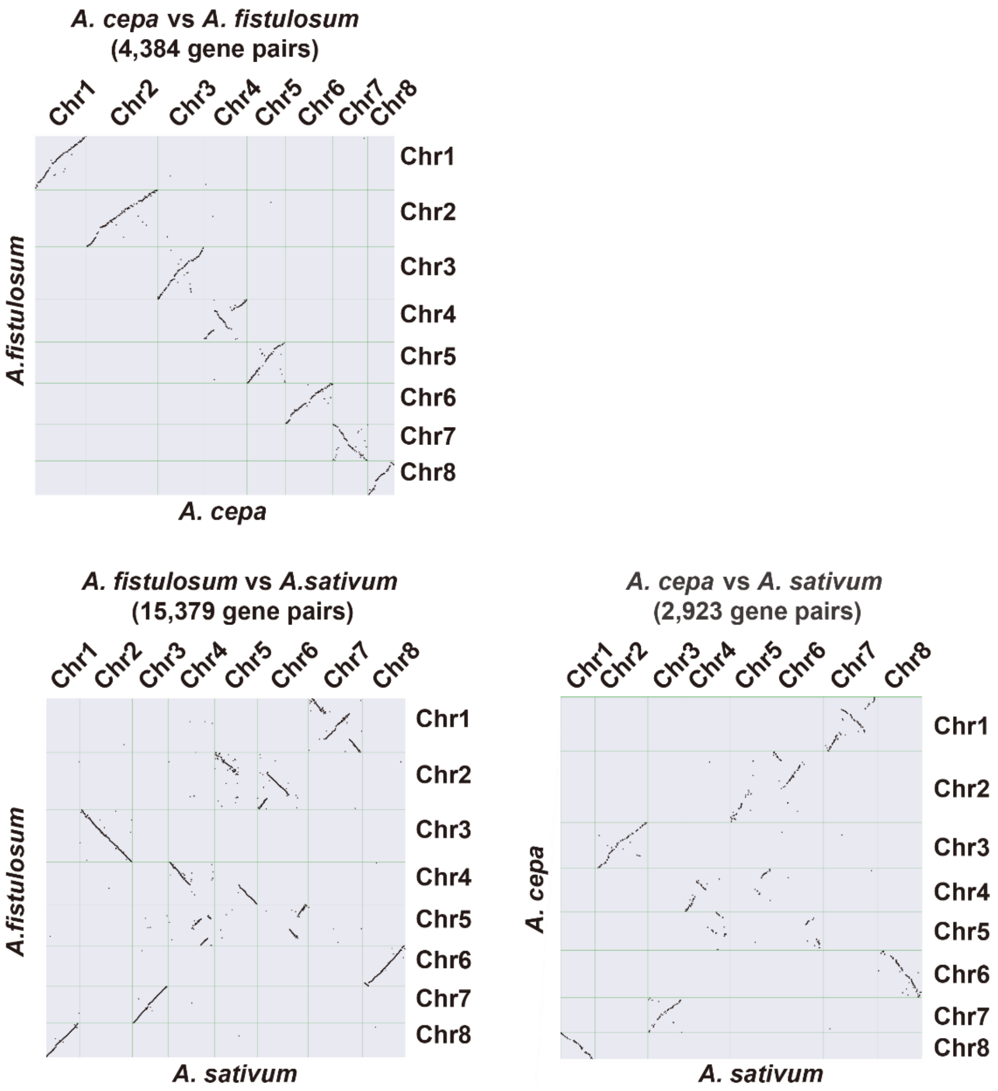

Supplementary Fig. 6. High genomic collinearity among three *Allium* crops.

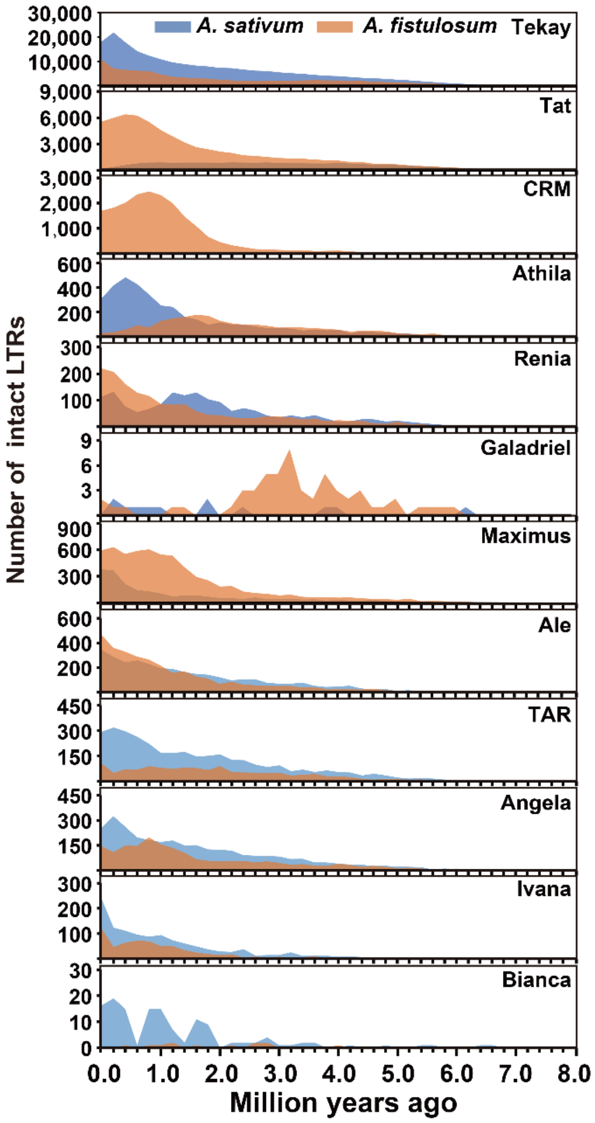

Supplementary Fig. 7. Average age of individual LTR families in *A. fistulosum* and *A. sativum*. Source data are provided as a Source Data file.

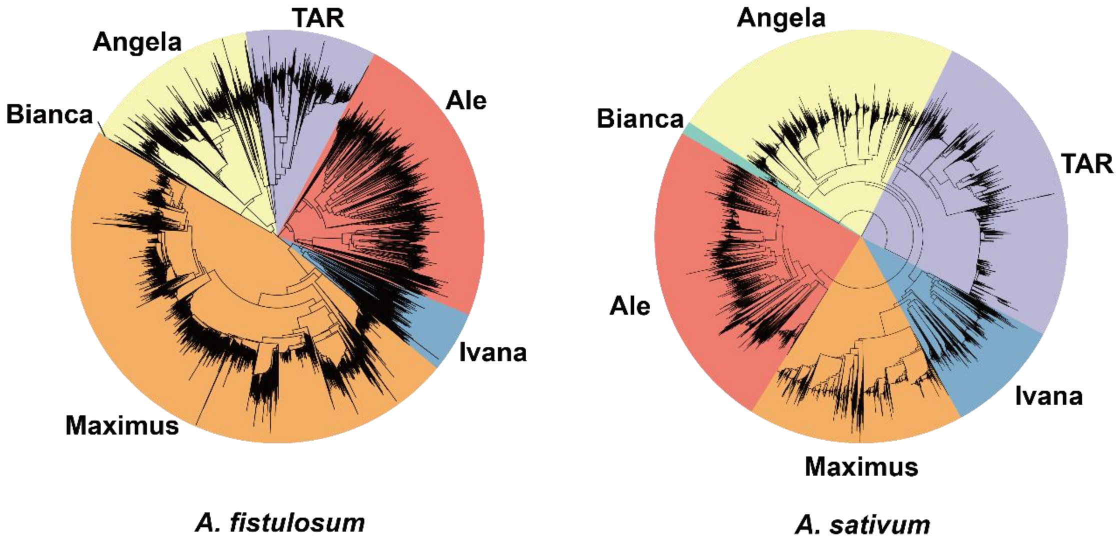

Supplementary Fig. 8. Phylogenetic relationships of *Copia* LTR-retrotransposons identified in *A. fistulosum* and *A. sativum* genomes.

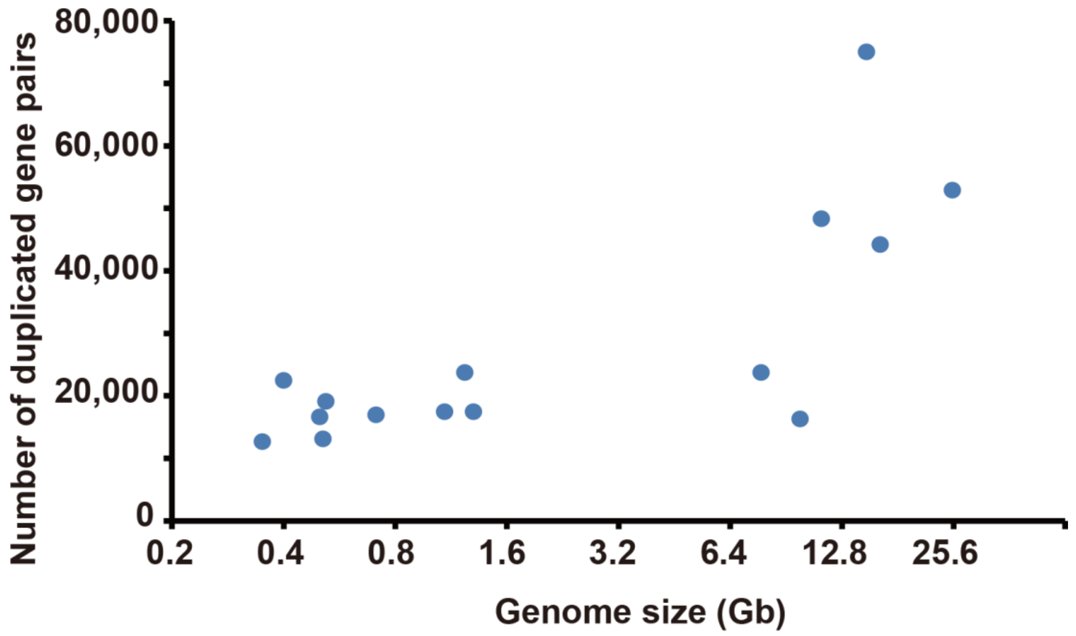

Supplementary Fig. 9. DSD genes contribute to gene expansions. Source data are provided as a Source Data file.

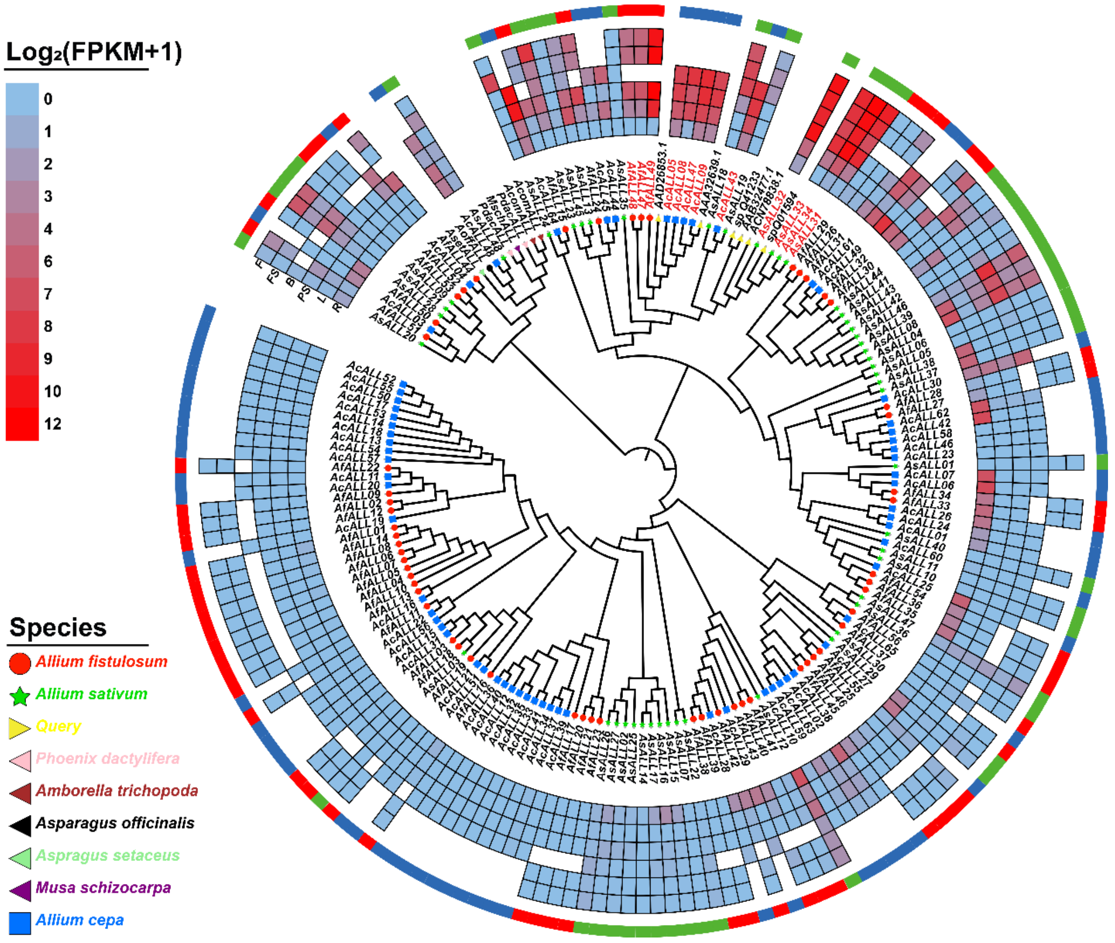

Supplementary Fig. 10. Phylogenetic tree of *ALLs* from eight genomes. The outer rings highlight the genes from *A. fistulosum* (red color), *A. cepa* (blue color), and *A. sativum* (green color), The heatmap exhibited the gene expressions in different tissues: R, roots; L, leaves; B, bulbs; PS, pseudo-stems; FS, floral stalks; F, flowers. Source data are provided as a Source Data file.

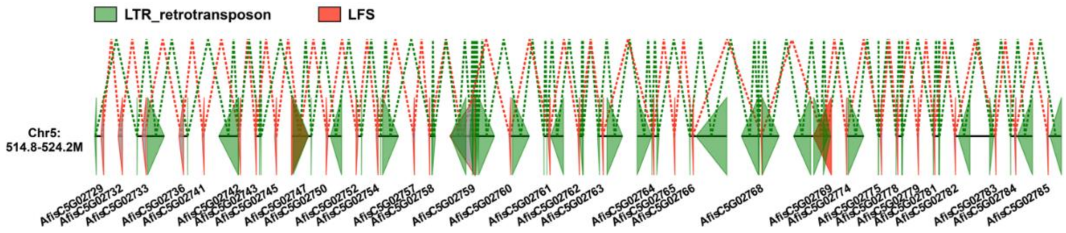

Supplementary Fig. 11. Contributions of LTRs to the expansion of LFS genes.

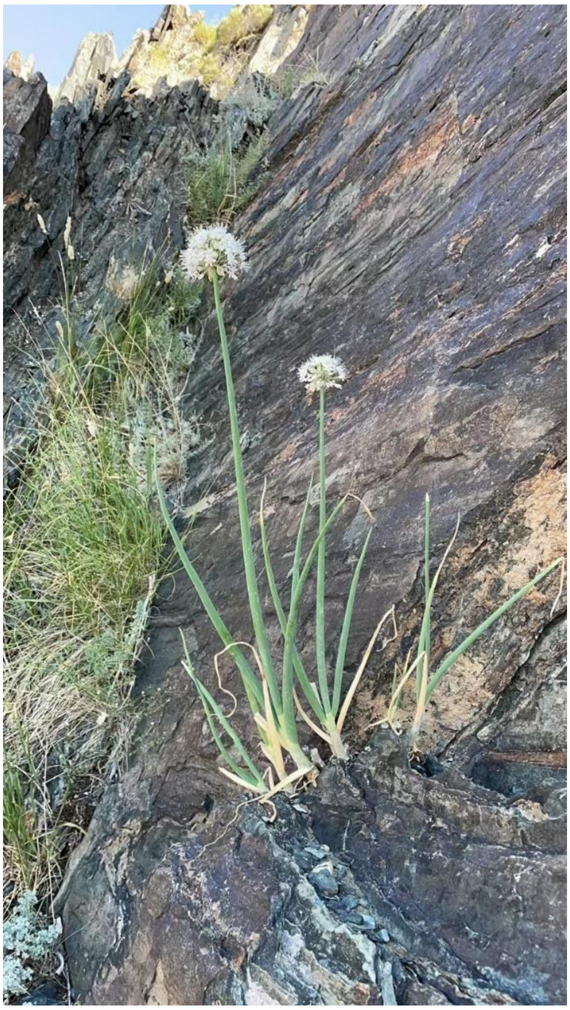

Supplementary Fig. 12. The harsh habitat of *A. altaicum* in Altaicum moutain. The picture was taken in Altaicum Moutain, Xinjiang, China (89.44E, 47.05N).

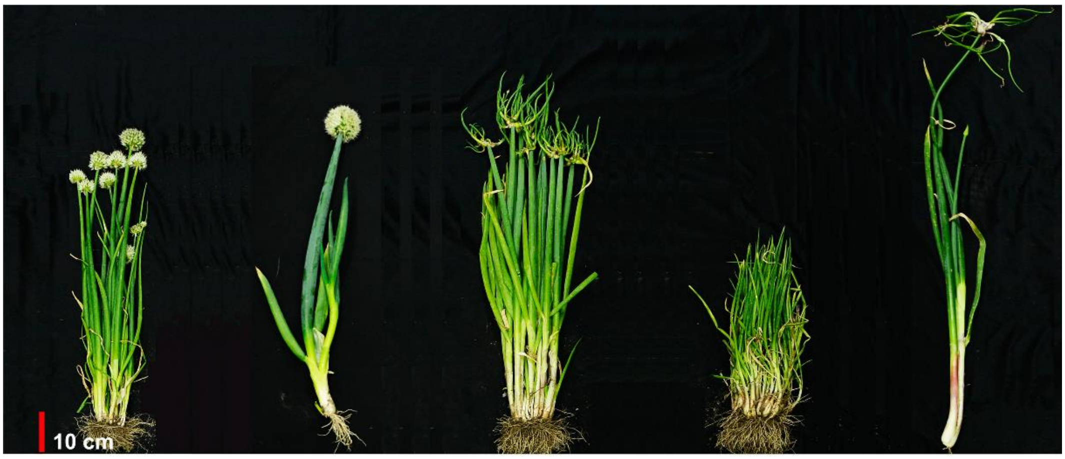

Supplementary Fig. 13. Phenotypes of the partial re-sequenced *Allium* accessions. From left to right, bunching onion, Welsh onion, storey onion, shallot, Chinese red onion.

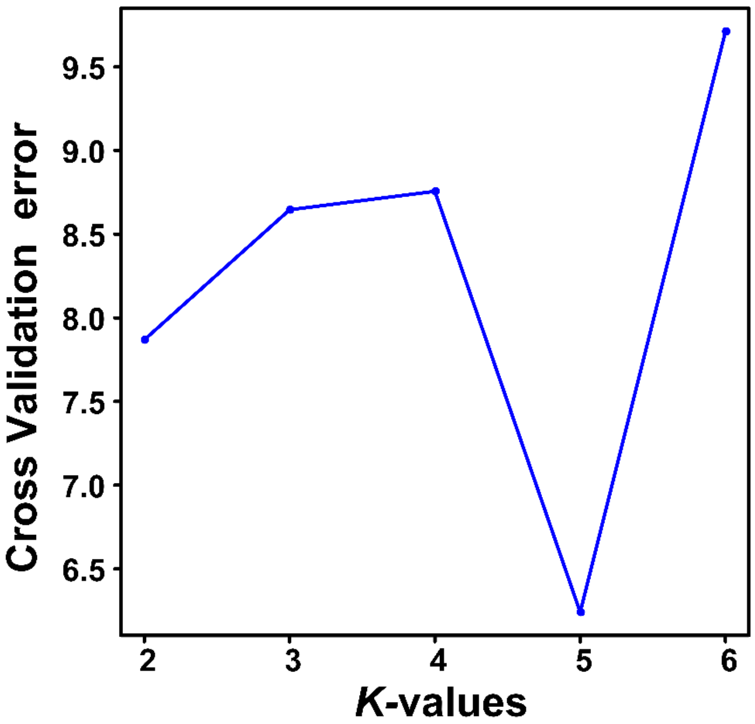

Supplementary Fig. 14. Cross-validation plot demonstrating the effect of different *K*-values on the cross-validation error. Source data are provided as a Source Data file.

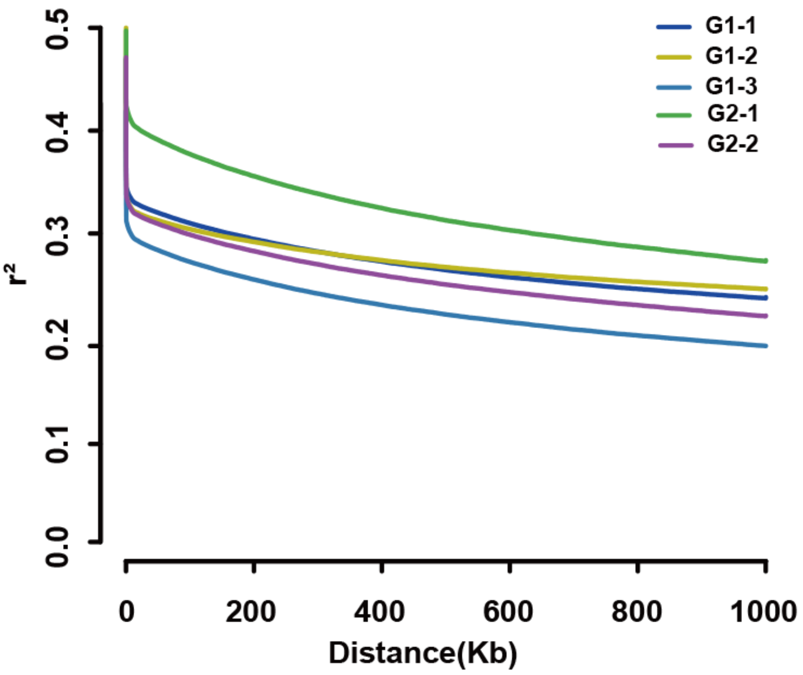

Supplementary Fig. 15. Genome-wide average LD decay estimated from five subgroups.

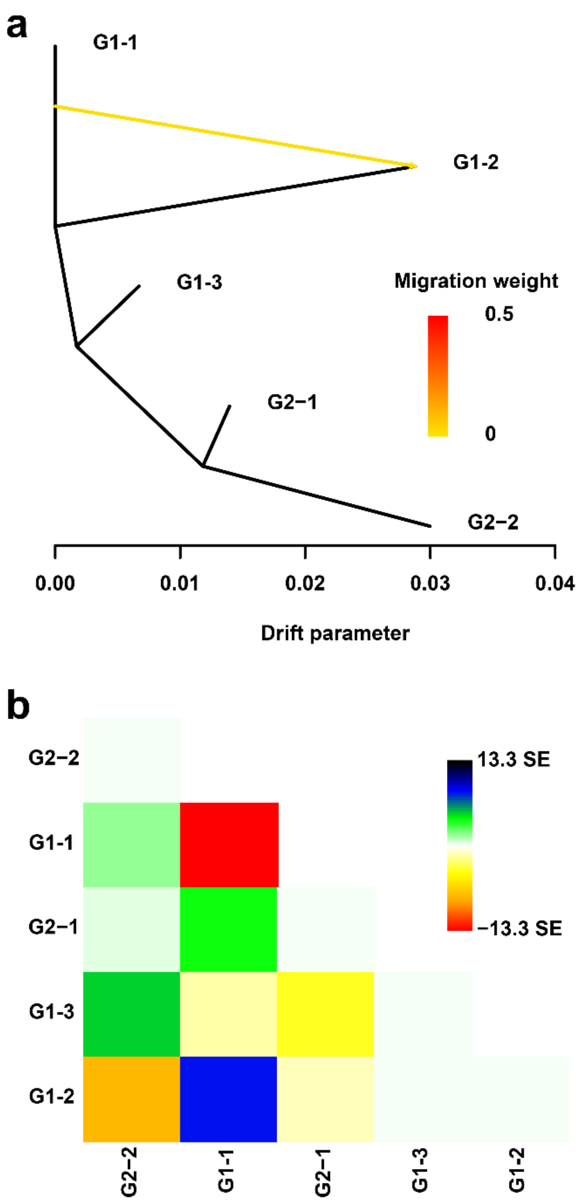

Supplementary Fig. 16. Gene flows of different *A. fistulosum* subgroups. a, Trees inferred by TreeMix. **b**, Residual fit, Plotted is the residual fit from the maximum likelihood tree.

	The aliphatic pathway																		The indolic and benzenic pathways								Co-substrate pathways				
	BCAT4	*BAT5*	*MAM1;MAM3*	*IPMI LSU1*	*IPMI SSU2; IPMI SSU3*	*IPMDH1;IPMDH3*	*BCAT3*	*CYP79F1;CYP79F2*	*CYP83A1*	*GSTF11*	*GSTU20*	*GGP1*	*SUR1*	*UGT74C1*	*SOT17; SOT18*	*FMO*	*APO*	*GS-OH*	*CYP79A2;B2;B3*	*CYP83B1*	*GSTF9;GSTF10*	*GGP1*	*SUR1*	*37-UGT74B1*	*SOT17; SOT16*	*CYP81F2*	*APK1;APK2*	*GSH1_PAD2*	*CHY1*	*AAO4*	*BZO1*
Arabidopsis thaliana	6	2	4	1	3	3	6	2	2	2	10	5	8	4	3	13	2	16	4	2	2	5	8	1	3	15	4	1	3	4	6
Allium sativum	5	1	2	2	0	3	5	0	0	0	9	2	6	0	0	1	0	4	0	0	0	2	6	0	0	3	2	4	5	2	2
Allium fistulosum	6	1	1	1	1	1	6	0	0	0	6	2	6	0	0	3	0	7	0	0	0	2	6	0	0	1	2	6	6	2	1
Allium cepa	5	1	1	1	1	1	5	0	0	0	7	2	6	0	0	3	0	5	0	0	0	2	6	0	0	1	3	5	5	2	1
Asparagus officinalis	3	1	0	1	0	1	3	0	0	0	3	3	3	0	0	2	0	4	0	0	0	3	3	0	0	0	2	1	3	0	0
Asparagus setaceus	4	1	3	4	1	1	4	0	0	0	6	3	2	0	0	2	0	7	0	0	0	3	2	0	0	1	2	1	3	2	2
Phalaenopsis equestris	3	1	2	1	1	1	3	0	0	0	8	1	0	0	0	3	0	4	0	0	0	1	0	0	0	4	3	1	1	0	1
Dendrobium officinale	6	2	4	1	2	1	6	0	0	0	5	1	1	0	0	2	0	2	0	0	0	1	1	0	0	2	9	3	2	1	1
Apostasia shenzhenica	3	1	2	1	1	1	3	0	0	0	2	1	0	0	0	1	0	1	0	0	0	1	0	0	0	0	2	1	1	0	1
Ananas comosus	3	1	1	2	1	1	3	0	0	0	3	2	1	0	0	1	0	7	0	0	0	2	1	0	0	1	3	1	1	3	2
Oryza sativa	5	1	2	1	1	1	5	0	0	0	0	1	5	2	0	1	0	3	1	0	0	1	5	0	0	1	3	2	1	7	2
Phoenix dactylifera	2	1	1	2	2	1	2	0	0	0	7	3	1	1	1	3	0	2	1	0	0	3	1	0	1	11	4	2	1	2	6
Musa schizocarpa	4	1	1	1	1	1	4	0	0	2	4	2	2	1	0	1	0	0	0	0	2	2	2	0	0	1	4	2	3	1	4
Amborella trichopoda	3	1	1	2	1	1	3	0	0	1	3	0	1	0	0	2	0	0	0	0	1	0	1	0	0	0	1	2	2	5	2

Supplementary Fig. 17. The number of each glucosinolates-related gene in 14 genomes.

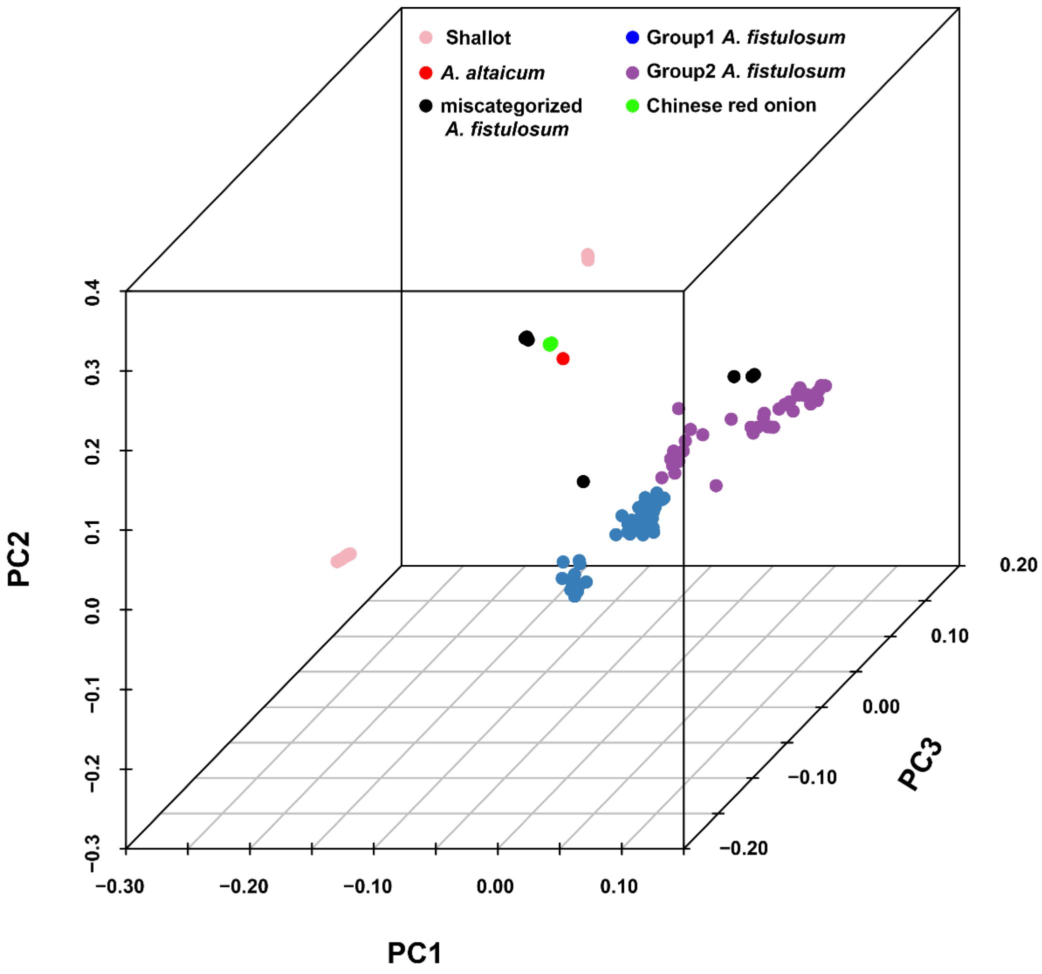

Supplementary Fig. 18. PCA analysis of 135 *Allium* accessions.
