# Supplementary material for: Chromosome-level genome assembly of bunching onion illuminates genome evolution and flavor formation in Allium crops
Source: Nat Commun. 2022 Nov 5;13:6690. doi: 10.1038/s41467-022-34491-3 (PMC9637129; doi:10.1038/s41467-022-34491-3)
Supplement: Supplementary file 2 — Description of Additional Supplementary Files [file 41467_2022_34491_MOESM2_ESM.pdf]

Description of Additional Supplementary Files

File Name: Supplementary Data 1
Description: Summary of the identified genes involved in sulfoxide biosynthesis in *Allium fistulosum*.

File Name: Supplementary Data 2
Description: Summary of the identified genes involved in sulfoxide biosynthesis in *Allium Cepa*.

File Name: Supplementary Data 3
Description: Summary of the identified genes involved in sulfoxide biosynthesis in *Allium Sativum*.

File Name: Supplementary Data 4
Description: Detailed information on accessions used for re-sequencing.
